# Cell-Type-Specific Sensorimotor Processing in Striatal Projection Neurons during Goal-Directed Behavior

**DOI:** 10.1016/j.neuron.2015.08.039

**Published:** 2015-10-21

**Authors:** Tanya Sippy, Damien Lapray, Sylvain Crochet, Carl C.H. Petersen

**Affiliations:** 1 Laboratory of Sensory Processing, Brain Mind Institute, Faculty of Life Sciences, École Polytechnique Fédérale de Lausanne (EPFL), Lausanne CH-1015, Switzerland

## Abstract

Goal-directed sensorimotor transformation drives important aspects of mammalian behavior. The striatum is thought to play a key role in reward-based learning and action selection, receiving glutamatergic sensorimotor signals and dopaminergic reward signals. Here, we obtain whole-cell membrane potential recordings from the dorsolateral striatum of mice trained to lick a reward spout after a whisker deflection. Striatal projection neurons showed strong task-related modulation, with more depolarization and action potential firing on hit trials compared to misses. Direct pathway striatonigral neurons, but not indirect pathway striatopallidal neurons, exhibited a prominent early sensory response. Optogenetic stimulation of direct pathway striatonigral neurons, but not indirect pathway striatopallidal neurons, readily substituted for whisker stimulation evoking a licking response. Our data are consistent with direct pathway striatonigral neurons contributing a “go” signal for goal-directed sensorimotor transformation leading to action initiation.

**Video Abstract:**

## Highlights

• Membrane potential recordings of striatal projection neurons in behaving mice
• Detected whisker stimuli evoke large sensorimotor responses in dorsolateral striatum
• Early sensory-evoked depolarization in striatonigral but not striatopallidal neurons
• Optogenetic substitution by striatonigral but not striatopallidal neurons

## Teaser

Sippy et al. measure cell-type-specific membrane potential dynamics in the striatum of head-restrained mice performing a simple goal-directed sensorimotor transformation. Their results are consistent with direct pathway striatonigral neurons delivering a fast “go” signal for action initiation.

## Introduction

A key function of the brain is to interpret incoming sensory information in the context of learned associations in order to guide adaptive behavior. However, the precise neuronal circuits and causal mechanisms underlying goal-directed sensorimotor transformations remain to be clearly defined for the mammalian brain. The basal ganglia are thought to be involved in action initiation and selection (Alexander and Crutcher, 1990; Graybiel et al., 1994; Grillner et al., 2005; Jin and Costa, 2010; Stephenson-Jones et al., 2011), and their dysfunction is associated with sensorimotor disorders, including Parkinson’s disease (Albin et al., 1989; DeLong, 1990; Kravitz et al., 2010). The input layer of the basal ganglia, the striatum, receives glutamatergic inputs from various cortical regions and the thalamus, as well as a significant dopaminergic projection, making this structure well-suited for integration of sensory input with reward signaling to produce appropriate motor output.

The vast majority of neurons in the striatum are GABAergic striatal projection neurons (SPNs). The SPNs can be subdivided according to their distinct long-range axonal projection patterns that correlate with differential gene expression (Gerfen et al., 1990; Bateup et al., 2010; Gerfen et al., 2013). The direct-pathway striatonigral neurons (dSPNs) expressing D1 receptors project to the substantia nigra and are often considered to form part of a “go” signaling pathway for action initiation, whereas the indirect pathway striatopallidal neurons (iSPNs) expressing D2 and A2A receptors project to the external segment of the globus pallidus and are thought to participate in “no go” signals (Durieux et al., 2009; Kravitz et al., 2012; Tai et al., 2012; Freeze et al., 2013). However, recent studies have failed to detect differences in the activity patterns of dSPNs versus iSPNs during task performance (Cui et al., 2013), questioning the validity of “go” and “no go” roles for these pathways.

Here, we investigate the role of the striatum in a simple sensorimotor task in which mice learn to lick for water reward in response to a single brief whisker deflection (Sachidhanandam et al., 2013). Because SPNs in vivo characteristically have low action potential firing rates (Wilson and Groves, 1981; Reig and Silberberg, 2014), we used whole-cell recordings to study both subthreshold and suprathreshold membrane potential (V_m_) activity of these neurons. Our recordings revealed strong task-related V_m_ dynamics in the dorsolateral striatum, with larger depolarizations on hit trials than miss trials. Interestingly, this activity differed substantially between the direct pathway striatonigral neurons and the iSPNs, with a fast transient excitation specifically in dSPNs. Optogenetic stimulation of dSPNs during task performance was consistent with brief excitation of the direct pathway playing a causal role in the sensorimotor transformation.

## Results

**Figure 1:**
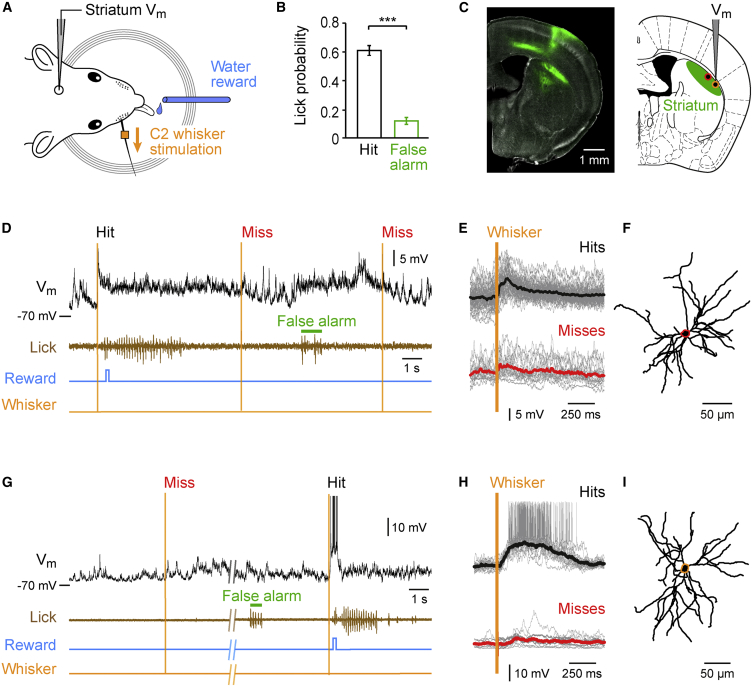
V_m_ Recordings from Identified Neurons in the Dorsolateral Striatum of Behaving Mice (A) Mice were head-restrained above an electromagnetic coil. Metal particles were placed on the right C2 whisker, which was deflected with a 1 ms current pulse delivered to the electromagnetic coil at random time intervals (6–10 s) without any preceding cue. Mice learned to lick the spout within 1 s of the whisker stimulus in order to receive a water reward. To control for random licking, stimulation trials were interleaved with catch trials in which no stimulation was given. (B) Mice learned the task over ∼1 week of training, reaching stable performance with hit rates (black) significantly higher than false alarm rates (green). Data are shown for performance during the electrophysiological recordings (n = 30 cells recorded across 25 mice, p = 1.7 × 10^−5^). (C) An anterograde tracer, AAV2-Synapsin-GFP, was injected into the left C2 barrel column showing prominent axonal innervation of left dorsolateral striatum (n = 3 mice) (left). Schematic coronal section showing the area of striatum (green) targeted for whole-cell recordings during the detection task (right). Color-coded circles show locations of the two example cells in (D)–(F) (red) and (G)–(I) (orange). (D) Example trace showing subthreshold V_m_ activity in an SPN during the detection task. V_m_ (black) of the neuron was recorded simultaneously with measurement of licking (brown) from the piezo-film attached to the reward spout. Licking within the 1 s reward window after C2 whisker stimulus (orange) opened a valve to deliver water reward (blue). (E) V_m_ of the SPN shown in (D), for all hit trials (black average, gray individual, n = 49 trials) and miss trials (red average, gray individual, n = 24 trials). (F) Dendritic structure of the recorded neuron. (G) V_m_ of a neuron with suprathreshold task-related activity. (H) Average traces from this neuron showing all hit (n = 21) and miss (n = 10) trials. (I) Dendritic structure of this neuron. Values are mean ± SEM. ^∗∗∗^p < 0.001. See also Figure S1.

We trained head-restrained mice to perform a simple goal-directed sensorimotor transformation in order to correlate behavioral performance with neuronal activity in the dorsolateral striatum. In our task, we delivered single 1-ms-duration deflections to the C2 whisker and trained mice to report detected stimuli by licking a reward spout (Figures 1A and S1) (Sachidhanandam et al., 2013). After mice were well-trained, we obtained whole-cell V_m_ recordings in the dorsolateral striatum during task performance (hit rate 60.9% ± 3.5%, false alarm rate 11.9% ± 2.8%, n = 30 cells) (Figure 1B). The dorsolateral striatum receives prominent excitatory glutamatergic input from primary somatosensory cortex (S1) (Figure 1C) (Wall et al., 2013; Reig and Silberberg, 2014), and S1 cortex is known to play a causal role in performance of this detection task (Sachidhanandam et al., 2013).

The whole-cell recording technique provides information about incoming subthreshold postsynaptic potentials and action potential output, which is particularly useful in brain regions dominated by neurons that have low firing rates. We recorded from 30 SPNs in 25 mice, and each neuron showed obvious task-related V_m_ dynamics. There was substantial diversity across different recordings, with many neurons (n = 24 cells) showing mostly subthreshold V_m_ changes (Figures 1D–1F) and a minority of neurons (n = 6 cells) firing task-related action potentials at high rates (Figures 1G–1I). Biocytin labeling introduced through the whole-cell recording pipette allowed anatomical identification of 27 out of 30 cells as SPNs, with their characteristic spiny morphology. The three other recorded neurons were also considered as SPNs, since they had similar electrophysiological properties to the identified cells, and these properties are not consistent with any other known striatal cell type.

### V_m_ of SPNs during Task Performance

**Figure 2:**
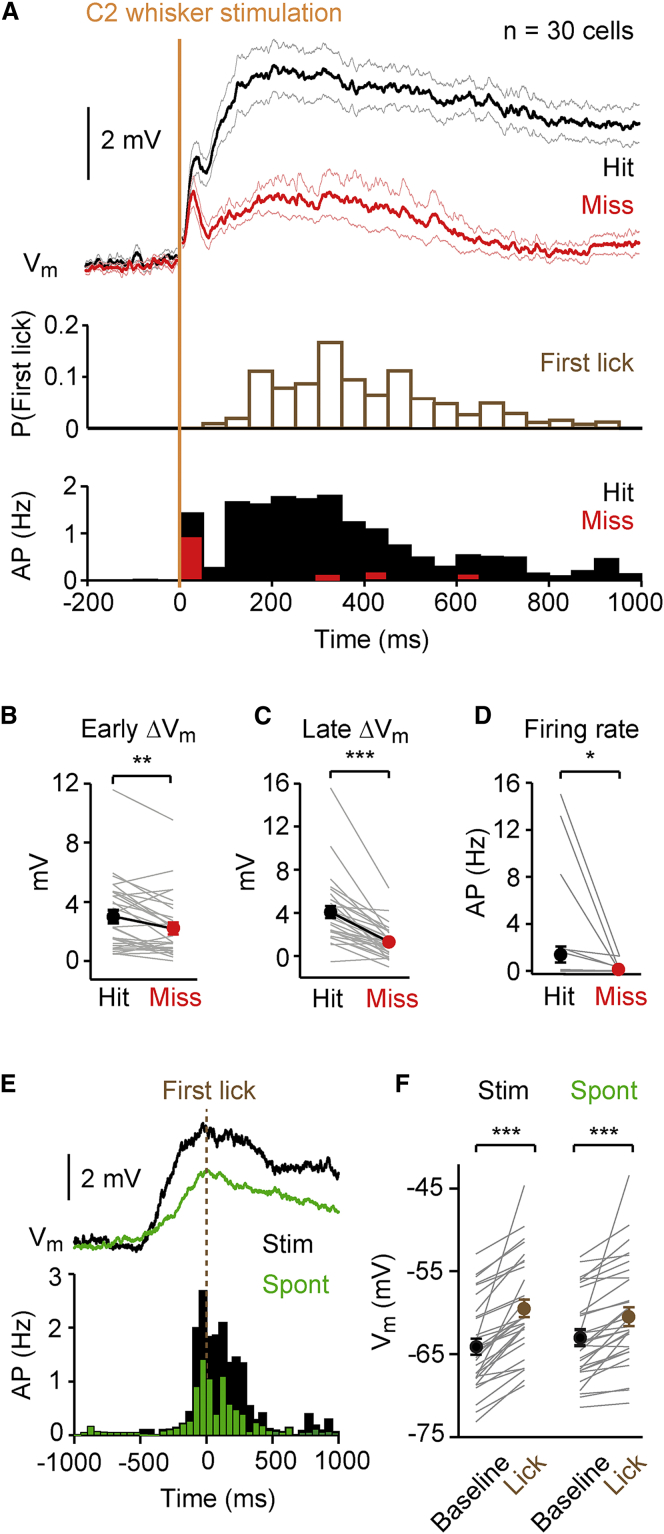
V_m_ of Striatal Neurons Correlates with Behavioral Performance (A) Grand average V_m_ across all SPNs recorded in response to whisker stimulation during hit (black) and miss (red) trials (above). Lighter shaded lines represent SEM. The time of the first lick in response to whisker stimulation across all hit trials (middle). The PSTH of action potential firing during hit (black) and miss (red) trials (below). (B) The early ΔV_m_ (calculated as the peak depolarization in the first 50 ms after whisker stimulation relative to prestimulus baseline V_m_) was significantly larger during hit compared to miss trials (n = 30 cells, Wilcoxon signed rank test p = 0.0024). (C) Late ΔV_m_ (calculated as the mean change in V_m_ from baseline to a period 50–250 ms after whisker stimulation) was significantly larger during hit trials versus miss trials (n = 30 cells, Wilcoxon signed rank test p = 2.3 × 10^−6^). (D) The firing rate of SPNs was significantly higher during hit versus miss trials (n = 30 cells, Wilcoxon signed rank test p = 0.02). (E) Average V_m_ and PSTH of SPNs around the time of the first lick on hit trials (“Stim,” black) and unrewarded spontaneous licking (“Spont,” green). (F) The V_m_ depolarized significantly from baseline before the time of the first lick for both hits (“Stim,” n = 30 cells, Wilcoxon signed rank test p = 1.7 × 10^−6^) and unrewarded spontaneous licking (“Spont,” n = 30 cells, Wilcoxon signed rank test p = 8.5 × 10^−6^). Values are mean ± SEM. ^∗^p < 0.05; ^∗∗^p < 0.01; ^∗∗∗^p < 0.001. See also Figure S2.

Important information can be learned about the neuronal activity underlying the conversion of sensory signals into goal-directed motor output by comparing hit and miss trials. In our recordings from SPNs in the dorsolateral striatum, we found striking differences in V_m_ dynamics depending upon behavioral outcome (hit versus miss), both for individual neurons (Figures 1D–1I) and analyzed across the population of recorded neurons (n = 30 cells) (Figure 2A). The grand average V_m_ showed two obvious phases, an early transient depolarization and a later longer-lasting depolarization (Figure 2A). The peak of the early (0–50 ms) depolarizing sensory response was significantly larger in hit trials (hit 3.0 ± 0.4 mV, miss 2.2 ± 0.4 mV, n = 30 cells, Wilcoxon signed rank test p = 0.002) (Figure 2B). During the secondary late phase (50–250 ms after whisker stimulus), the average evoked V_m_ depolarization in the dorsolateral striatum was also significantly larger in hit trials (hit 4.0 ± 0.5 mV, miss 1.3 ± 0.3 mV, n = 30 cells, Wilcoxon signed rank test p = 2 × 10^−6^) (Figure 2C). Action potential firing was also increased in hit trials (hit 1.40 ± 0.68 Hz, miss 0.10 ± 0.05 Hz, n = 30 cells, Wilcoxon signed rank test p = 0.016) (Figure 2D).

We were curious if pre-stimulus differences in striatal V_m_ could account for behavioral performance. We therefore compared the pre-stimulus baseline V_m_, the fast fourier transform of the V_m_ and the correlation of the V_m_ with the local field potential recorded in S1 in the 2 s preceding each whisker stimulus. However, similar to results in S1 (Sachidhanandam et al., 2013), we did not find differences in the pre-stimulus V_m_ in the striatum comparing hit and miss trials (Figure S2).

The striatum is thought to be important for initiation and control of movement, leading us to question how the V_m_ of SPNs was modulated by licking during the behavioral task. We therefore aligned the V_m_ traces of SPNs with respect to the mouse’s first lick during both stimulus (hit) trials and unrewarded spontaneous licking (Figure 2E). The V_m_ was significantly depolarized from baseline with respect to licking during both hit trials and spontaneous licking (change in V_m_ from baseline on hit trials 4.8 ± 0.6 mV, n = 30 cells, Wilcoxon signed rank test p = 1.7 × 10^−6^; change in V_m_ from baseline for spontaneous licking 3.0 ± 0.7 mV, n = 30 cells, Wilcoxon signed rank test p = 8.5 × 10^−6^) (Figures 2E and 2F). The depolarization began hundreds of milliseconds before licking, and excitation of striatal neurons could therefore contribute to initiating the licking motor response.

### V_m_ Dynamics of Direct and Indirect Pathway SPNs

**Figure 3:**
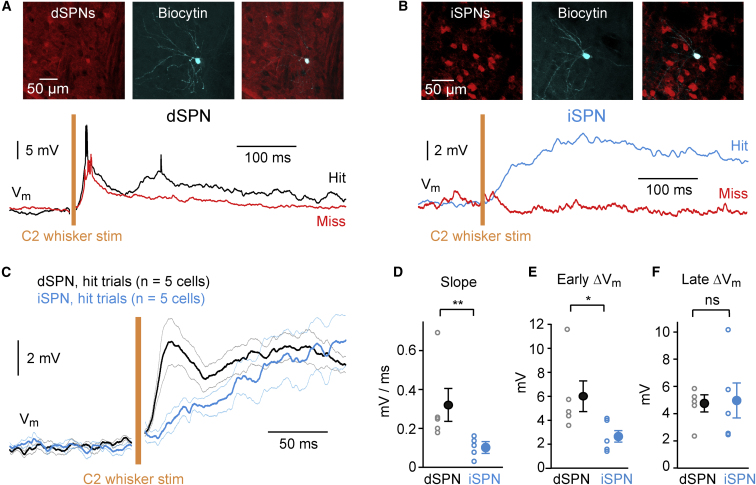
Cell-Type-Specific Sensorimotor Processing during the Detection Task (A) A D1-Cre mouse was crossed with a LSL-tdTomato mouse, driving expression of tdTomato in dSPNs (upper left). The recorded neuron was filled with biocytin and stained with Alexa-647 (upper middle). The labeled neuron was considered as a dSPNs because the Alexa-647 signal co-localized with tdTomato (upper right). Average hit and miss traces from the example positively identified dSPN (below). (B) iSPNs express tdTomato in an A2A-Cre × LSL-tdTomato mouse (upper left). The recorded neuron was filled with biocytin and stained with Alexa-647 (upper middle). The biocytin stain co-localized with tdTomato defining this neuron as an iSPN (upper right). Average hit and miss traces (below) from the example positively identified iSPN. (C) Grand average V_m_ of hit trials across all positively identified dSPNs (black, n = 5 cells) and iSPNs (blue, n = 5 cells), showing an early sensory response specifically in dSPNs. Lighter shaded lines represent SEM. (D) The slope of the early response was significantly larger in dSPNs versus iSPNs (n = 5 of each cell type, Wilcoxon Mann-Whitney two-sample rank test, p = 0.008). (E) The amplitude of the early response (Early ΔV_m_), was significantly larger for dSPNs versus iSPNs (n = 5 of each cell type, Wilcoxon Mann-Whitney two-sample rank test, p = 0.03). (F) The late response (Late ΔV_m_) was not significantly different in dSPNs versus iSPNs (n = 5 of each cell type, Wilcoxon Mann-Whitney two-sample rank test, p = 1). Values are mean ± SEM. ^∗^p < 0.05; ^∗∗^p < 0.01; ns, non-significant. See also Figure S3.

In a subset of our experiments, we were able to unambiguously identify the type of SPN through post hoc histology. The intracellular pipette solution contained biocytin, allowing for colocalization of fluorescent biocytin staining with tdTomato fluorescence in D1-Cre mice (for dSPNs) and D2-Cre or A2A-Cre mice (for iSPNs) crossed with LSL-tdTomato reporter mice (Figures 3A and 3B) (Madisen et al., 2010; Gerfen et al., 2013). A neuron was identified as a dSPN or iSPN only if the biocytin-filled neuron also expressed tdTomato. Neurons that were filled with biocytin but did not express tdTomato were not included in the analysis in order to avoid incorporating false negatives into our dataset.

In this subset of positively defined subtypes of SPNs, it was apparent that the early (0–50 ms) response was much more pronounced in dSPNs compared to iSPNs (Figures 3C and S3). Quantification of the slope of the early sensory response in hit trials revealed it to be significantly faster in dSPNs (dSPN 0.32 ± 0.10 mV.ms^−1^, n = 5 cells; iSPN 0.10 ± 0.02 mV/ms, n = 5 cells; Wilcoxon Mann-Whitney two-sample rank test p = 0.008) (Figure 3D). The amplitude of the early response in hit trials was also significantly larger in dSPNs when compared to iSPNs (dSPN 6.0 ± 1.4 mV, n = 5 cells; iSPN 2.6 ± 0.5 mV, n = 5 cells; Wilcoxon Mann-Whitney two-sample rank test p = 0.03) (Figure 3E).

We also compared the late phase (50–250 ms post-stimulus) in dSPNs and iSPNs. We found that during the late phase, the two neuron types were equally depolarized on hit trials (dSPNs 4.6 ± 0.6 mV, n = 5 cells; iSPNs 5.1 ± 1.4 mV, n = 5 cells; Wilcoxon Mann-Whitney two-sample rank test, p = 1) (Figure 3F). Our data therefore reveal a strong rapid transient sensory-evoked depolarization specifically in dSPNs, which could contribute a “go” signal to initiate licking behavior.

### Optogenetic Activation of the Direct and Indirect Pathways

**Figure 4:**
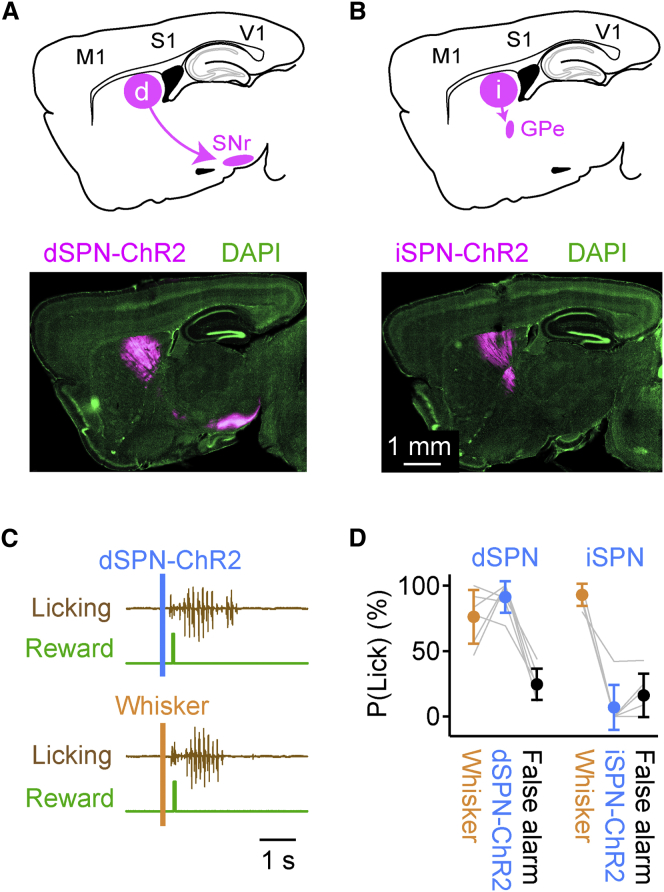
Optogenetic Stimulation of dSPNs, but Not iSPNs, Substitutes for Whisker Stimulation during the Detection Task (A) Schematic sagittal section showing the direct-pathway projection (dSPNs) from striatum to SNr (above). Fluorescence image of a sagittal section from a D1-Cre mouse injected in the dorsolateral striatum with AAV-DIO-ChR2-YFP (below). Antibody staining for YFP (magenta) in axons shows the projection of dSPNs to SNr. DAPI staining of cell nuclei shown in green. (B) Schematic of sagittal section showing indirect-pathway (iSPNs) projecting from striatum to the external segment of the globus pallidus (GPe). An A2A-Cre mouse was infected with AAV-DIO-ChR2-YFP in the dorsolateral striatum and antibody staining for YFP (magenta) shows the iSPN projection to GPe. (C) On the transfer test day, 50-ms flashes of blue light delivered into the dorsolateral striatum (above) were interleaved with whisker stimuli (below) and trials without stimulation. As shown in these example traces, blue light activation of dSPNs was able to drive licking. (D) Performance of dSPN-ChR2 mice in response to a 50-ms blue light stimulus on the transfer test day was similar to whisker stimulus trials (n = 6 mice, Kruskal-Wallis test followed by Student-Newman-Keuls test, p > 0.05) and significantly above the false alarm rate (n = 6 mice, Kruskal-Wallis test followed by Student-Newman-Keuls test, p < 0.005). However, performance of iSPN-ChR2 mice with 50-ms blue light was significantly lower than for whisker stimuli (n = 6 mice, Kruskal-Wallis test followed by Student-Newman-Keuls test, p < 0.005), and similar to the false alarm rate in these animals (n = 6 mice, Kruskal-Wallis test followed by Student-Newman-Keuls test, p > 0.05). Values are mean ± SD. See also Figure S4.

In order to test our hypothesis that the early response in dSPNs might contribute to initiate movement, we sought to activate this pathway specifically in the context of the detection task. Toward this goal, we made use of an optogenetic approach. We injected Cre-dependent adeno-associated viral vectors encoding channelrhodopsin-2 (ChR2) linked to YFP into the dorsolateral striatum of either D1-Cre or A2A-Cre mice, thereby expressing the opsin specifically in either dSPNs or iSPNs, respectively. Antibody staining of YFP revealed direct pathway dSPN axons projecting to substantia nigra pars reticulata (SNr) in D1-Cre mice (Figure 4A, and indirect-pathway iSPN axons innervating the external segment of globus pallidus (GPe) in A2A-Cre mice (Figure 4B). The mice were trained to detect whisker stimulation following our standard training procedures, except for the addition of blue background light. After stable whisker detection performance was reached, on a given transfer-test day, the first blue light flashes were introduced to the striatal neurons via an optical fiber. The optogenetic stimuli (ranging from 5–500 ms in duration) were randomly interleaved with standard whisker stimulation trials and catch trials without whisker stimulation (Figure S4). Brief, single optogenetic stimuli delivered to dSPNs readily substituted for whisker stimulation and evoked robust licking (Figure 4C). Across all mice tested, the hit rate evoked by 50-ms ChR2 stimulation of dSPNs (91% ± 5%, n = 6 mice) was significantly higher than the false alarm rate (25% ± 5%, n = 6 mice, Kruskal-Wallis test followed by Student-Newman-Keuls test p < 0.005), and not different from the whisker stimulus evoked hit rate (76% ± 8%, n = 6 mice, Kruskal-Wallis test followed by Student-Newman-Keuls test p > 0.05) (Figure 4D). In contrast, optogenetic stimulation of iSPNs did not induce licking (Figures 4D and S4). The hit rate evoked by 50-ms ChR2 stimulation of iSPNs (7% ± 7%, n = 6 mice) was significantly lower than the whisker-stimulus-evoked hit rate (93% ± 3%, n = 6 mice, Kruskal-Wallis test followed by Student-Newman-Keuls test p < 0.005) and not significantly different from the false alarm rate (16% ± 7%, n = 6 mice, Kruskal-Wallis test followed by Student-Newman-Keuls test, p > 0.05). Therefore, only activation of dSPNs (and not iSPNs) reliably substituted for sensory stimulation during the detection task.

We also carried out the same optogenetic stimulation experiments in free-licking thirsty mice, which were not trained in the whisker detection task but which were trained to lick the spout in order to obtain water reward. In these highly motivated mice, stimulation of dSPNs, but not iSPNs, evoked licking (Figure S4). This suggests that the licking evoked by dSPN stimulation in the mice performing the whisker detection task relates more to a motor signal than a sensory signal.

## Discussion

By using the whole-cell recording technique, we characterized the V_m_ of dorsolateral SPNs in awake, behaving mice. We examined changes in subthreshold and suprathreshold activity during a sensorimotor task requiring motivation and found that the V_m_ of SPNs strongly correlates with behavioral performance. Only neurons of the direct striatonigral pathway exhibited a prominent early sensory response, and only optogenetic stimulation of the direct striatonigral pathway substituted for peripheral stimulation. Our results extend current knowledge of V_m_ dynamics of SPNs and lend support for a mechanism by which the direct pathway striatonigral neurons contribute to initiate movement in the context of motivation.

### V_m_ Measurements in Striatum and Cortex during the Detection Task

We previously measured V_m_ in the primary somatosensory cortex (S1) during the same detection task as used in this study (Sachidhanandam et al., 2013). It is therefore interesting to consider the similarities and differences between these closely related brain areas in their V_m_ dynamics evoked by the 1-ms deflection of the C2 whisker during task performance. In both cortex and striatum, we found biphasic depolarizing V_m_ responses consisting of an early sensory component and a later motor-related component. However, there were important qualitative and quantitative differences between the responses in cortex and striatum.

In layer 2/3 of S1 cortex, we found that the early sensory response is present in all neurons and does not differ between hit and miss trials (Sachidhanandam et al., 2013). In striatum, however, the early sensory response was specifically found in dSPNs (Figure 3) and was significantly larger in amplitude during hit trials (Figure 2). dSPNs in dorsolateral striatum receive strong input from whisker S1 (Wall et al., 2013; Reig and Silberberg, 2014), and corticostriatal input is likely to contribute importantly to the early sensory response in dSPNs. In future studies, it will therefore be important to determine if corticostriatal projection neurons in S1 provide differential excitation to SPNs on hit versus miss trials or whether this is a result of synaptic computation within the striatum or other neural circuit.

The late depolarization was larger in hit trials compared to miss trials both in striatum (Figure 2) and in layer 2/3 of S1 cortex (Sachidhanandam et al., 2013). However, the difference between the late depolarization in hits and misses is much larger in the dorsolateral SPNs compared to S1 cortex. The late depolarization (50–250 ms) follows the early sensory response but precedes licking motor output. Given that striatal V_m_ depolarized before both rewarded and spontaneous licking (Figure 2), the enhanced late phase in striatum on hit trials might, at least in part, be a motor-related signal, perhaps selectively promoting licking while inhibiting other motor output. Interestingly, the late depolarization was equally large in dSPNs and iSPNs, unlike the early sensory response (Figure 3). Whereas dSPNs receive stronger input from sensory cortex, iSPNs receive stronger input from motor cortex (Wall et al., 2013), and in future work, it will be interesting to investigate the differential cortical inputs to dSPNs and iSPNs during the early and late responses of hit trials.

### The Early Response as a “Go” Signal in Direct Pathway Striatal Neurons

We have shown that a brief excitatory signal in dSPNs correlates with (Figures 1, 2, and 3) and is sufficient for (Figure 4) task performance. We speculate that such a signal could arise from reward-feedback during task learning through dopamine-related synaptic plasticity at corticostriatal synapses, with D1 receptor signaling helping potentiate synaptic input from S1 onto dSPNs and D2 receptor signaling perhaps promoting synaptic depression in iSPNs (Kreitzer and Malenka, 2008; Gerfen and Surmeier, 2011; Tritsch and Sabatini, 2012; Kress et al., 2013; Shepherd, 2013; Shan et al., 2014). Transient excitation of dSPNs could contribute to initiating licking motor output through at least two different circuits downstream of the SNr (Figure S4). Increased dSPN firing will inhibit the tonically active GABAergic SNr neurons projecting to downstream brainstem motor regions, thus causing disinhibition and enhancing motor output (Grillner et al., 2005; Freeze et al., 2013). GABAergic neurons in the SNr also project to the thalamus, which therefore becomes disinhibited by the dSPN “go” signal. Increased thalamic activity could contribute to late cortical depolarization, known to correlate with and contribute to task performance (Sachidhanandam et al., 2013), which could then form part of a recurrent, positive feedback loop through striatal-thalamo-cortical circuits.

Beyond the initial 50-ms-duration “go” excitation signal in dSPNs, the two types of SPNs behaved in a very similar way, with both cell types depolarizing equally at late times during hit trials (Figure 3E). It may thus be the fast signaling of the whisker detection task that gives our experimental paradigm sufficient temporal precision to uncover the specific dSPN “go” signal, which might not have been resolved in previous measurements (Cui et al., 2013). It should be noted, however, that due to the sparse AP firing rates of SPNs, our study includes only a small number of SPNs with appreciable spiking, and further studies will therefore be important to better characterize cell-type-specific firing patterns of striatal neurons during diverse behaviors.

Our data are consistent with corticostriatal signals contributing to simple goal-directed sensorimotor transformation (Znamenskiy and Zador, 2013; Xiong et al., 2015), perhaps resulting from learning under guidance of dopamine signals evoked by whisker stimulation serving as a reward predictor (Schultz et al., 1997; Schultz, 1998; Cohen et al., 2012). To test these hypotheses, in future experiments it will be important to record and manipulate the activity of dopaminergic neurons and also to test whether corticostriatal input from the C2 barrel column in S1 undergoes learning-induced plasticity that is necessary and sufficient for task performance.

## Experimental Procedures

### Animal Preparation and Surgery

All experiments were carried out in accordance with protocols approved by the Swiss Federal Veterinary Office. For electrophysiological recordings, D1R-Cre, D2R-Cre, and A2AR-Cre mice (Gerfen et al., 2013) were crossed with Lox-STOP-Lox-tdTomato (LSL-tdTomato) reporter mice (Madisen et al., 2010). For optogenetic experiments, AAV-DIO-ChR2 virus was stereotactically injected into D1R-Cre or A2AR-Cre mice. A metal head-holder was implanted under anesthesia.

### Behavioral Training

Mice were trained to lick a water-reward spout in response to single 1-ms-duration deflections of the C2 whisker following previously described procedures (Sachidhanandam et al., 2013).

### Electrophysiology

Whole-cell patch-clamp recording electrodes (5–7 MΩ) were filled with an intracellular solution containing (in mM) 135 potassium gluconate, 4 KCl, 10 HEPES, 10 sodium phosphocreatine, 4 MgATP, and 0.3 Na_3_GTP (adjusted to pH 7.3 with KOH), to which 3 mg/ml biocytin was added. V_m_ was recorded using a Multiclamp 700B amplifier without injection of holding current and was not corrected for liquid junction potentials.

### Optogenetics

Blue light was delivered using a 300-μm-diameter optical fiber coupled to a 473 nm laser inserted into the brain directly above dorsolateral striatum.

### Data Analysis

Electrophysiological and behavioral data were analyzed in Matlab. Data are presented as mean ± SEM throughout the text and figures, except Figure 4D, which shows mean ± SD.

## Author Contributions

T.S., D.L., S.C., and C.C.H.P. designed the project and wrote the manuscript. T.S., D.L., and S.C. performed experiments and analyzed data.

## Supplemental Information

Document S1. Figures S1–S4 and Supplemental Experimental Procedures

Document S2. Article plus Supplemental Information
