# Supplementary figures and images for: Cell-Type-Specific Sensorimotor Processing in Striatal Projection Neurons during Goal-Directed Behavior
Source: Neuron. 2015 Oct 21;88(2):298–305. doi: 10.1016/j.neuron.2015.08.039 (PMC4622932; doi:10.1016/j.neuron.2015.08.039)

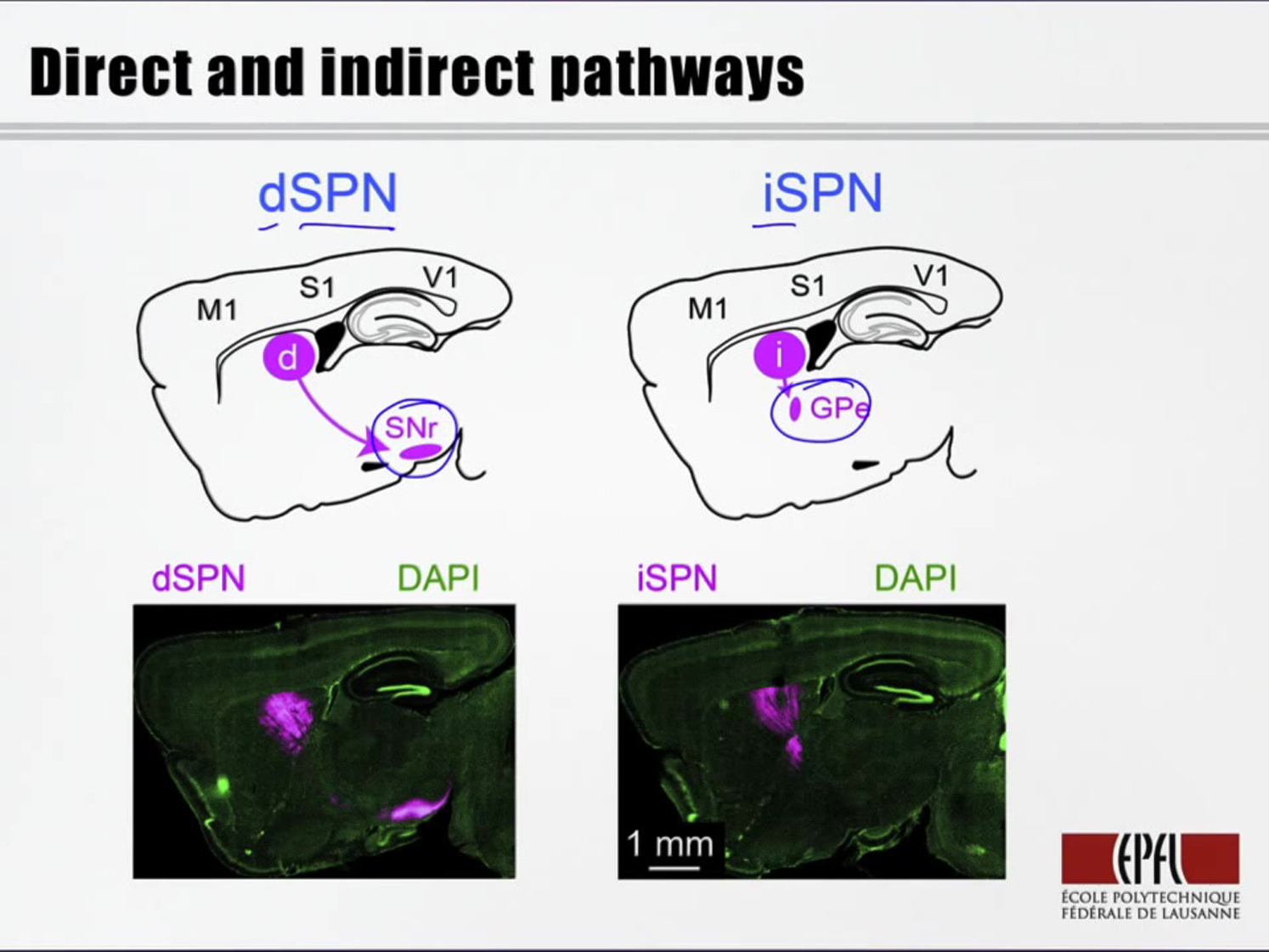

Supplement: Supplementary file 1 [file mmc3.jpg]
