# Supplementary material for: Cell-Type-Specific Sensorimotor Processing in Striatal Projection Neurons during Goal-Directed Behavior
Source: Neuron. 2015 Oct 21;88(2):298–305. doi: 10.1016/j.neuron.2015.08.039 (PMC4622932; doi:10.1016/j.neuron.2015.08.039)
Supplement: Document S1. Figures S1–S4 and Supplemental Experimental Procedures [file mmc1.pdf]

Neuron, Volume *88*

Supplemental Information

Cell-Type-Specific Sensorimotor Processing

in Striatal Projection Neurons

during Goal-Directed Behavior

Tanya Sippy, Damien Lapray, Sylvain Crochet, and Carl C. H. Petersen

Supplemental information

Cell-type-specific sensorimotor processing in striatal projection neurons during goal-directed behavior

Tanya Sippy, Damien Lapray, Sylvain Crochet
and Carl C. H. Petersen

Supplemental Information

Supplemental Figures S1-S4

Supplemental Experimental Procedures

Figure S1

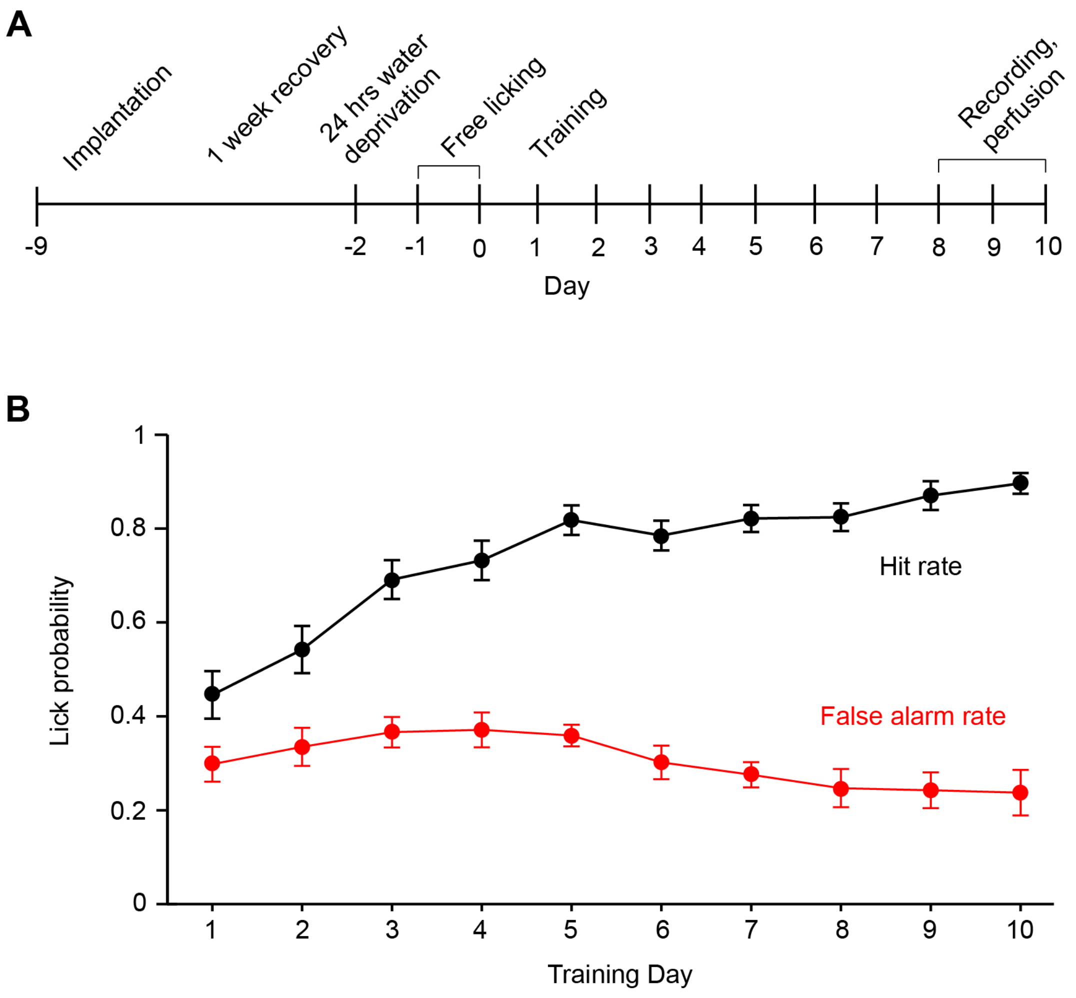

Figure S1. Behavioral training, related to Figure 1.

(A) Timeline of mouse preparation and behavioral training. Mice were implanted with a head-post and allowed to recover for 1 week. They were then water deprived for 24 hours and habituated to head restraint while being given free access to water through the reward spout ('free licking'). On subsequent days, mice were trained on the detection task until stable performance was achieved, usually by training day 8.

(B) Day-by-day behavioral performance curve for all mice (n = 28 mice, of which whole-cell recordings were obtained in 25 of these mice). Mice achieved a stable level of performance after approximately 1 week of training. Values are mean ± SEM.

Figure S2

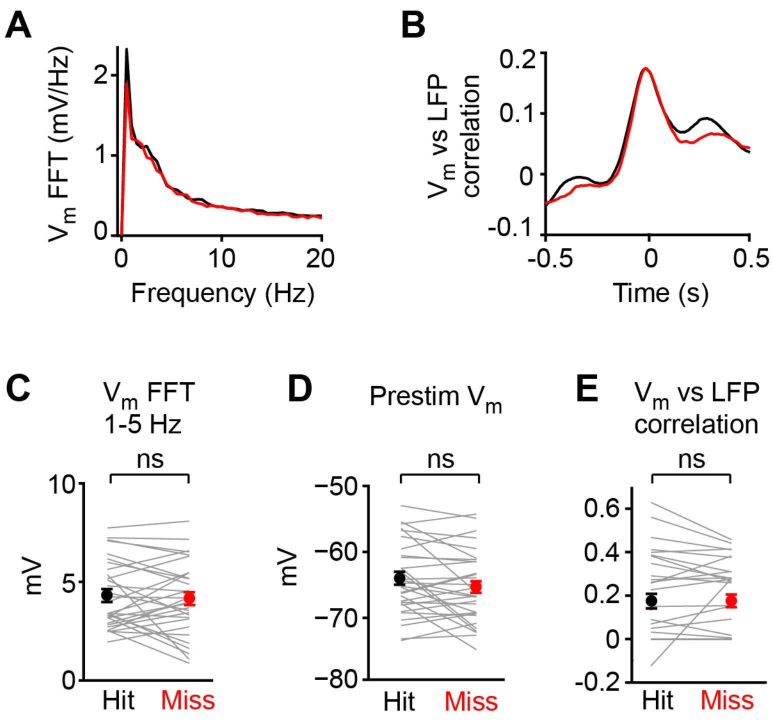

Figure S2. A comparison of pre-stimulus membrane potential revealed no difference between hit and miss trials, related to Figure 2.

(A) Grand average FFT of the V_m calculated for the 2 second period prior to each stimulation for hit (black) and miss (red) trials, averaged across n = 30 cells. Prestimulus V_m displayed prominent slow fluctuations in some striatal neurons, but this was not different in hit *vs* miss trials.

(B) Grand average (n = 21 cells) pre-stimulus cross-correlograms between V_m in striatum and LFP recorded in C2 barrel column for the 2 s preceding both hit (black) and miss (red trials).

(C) The prestimulus V_m FFT 1-5 Hz integral was not significantly different between hit and miss trials (n = 30 cells, Wilcoxon signed rank test p = 0.5).

(D) The prestimulus V_m was not significantly different between hit and miss trials (n = 30 cells, Wilcoxon signed rank test p = 0.5).

(E) The zero-time cross-correlation between striatal V_m and S1 LFP was not significantly different for hit *vs* miss trials (n = 21 cells, Wilcoxon signed rank test p = 0.3).

Values are mean ± SEM. ns = non-significant.

Figure S3

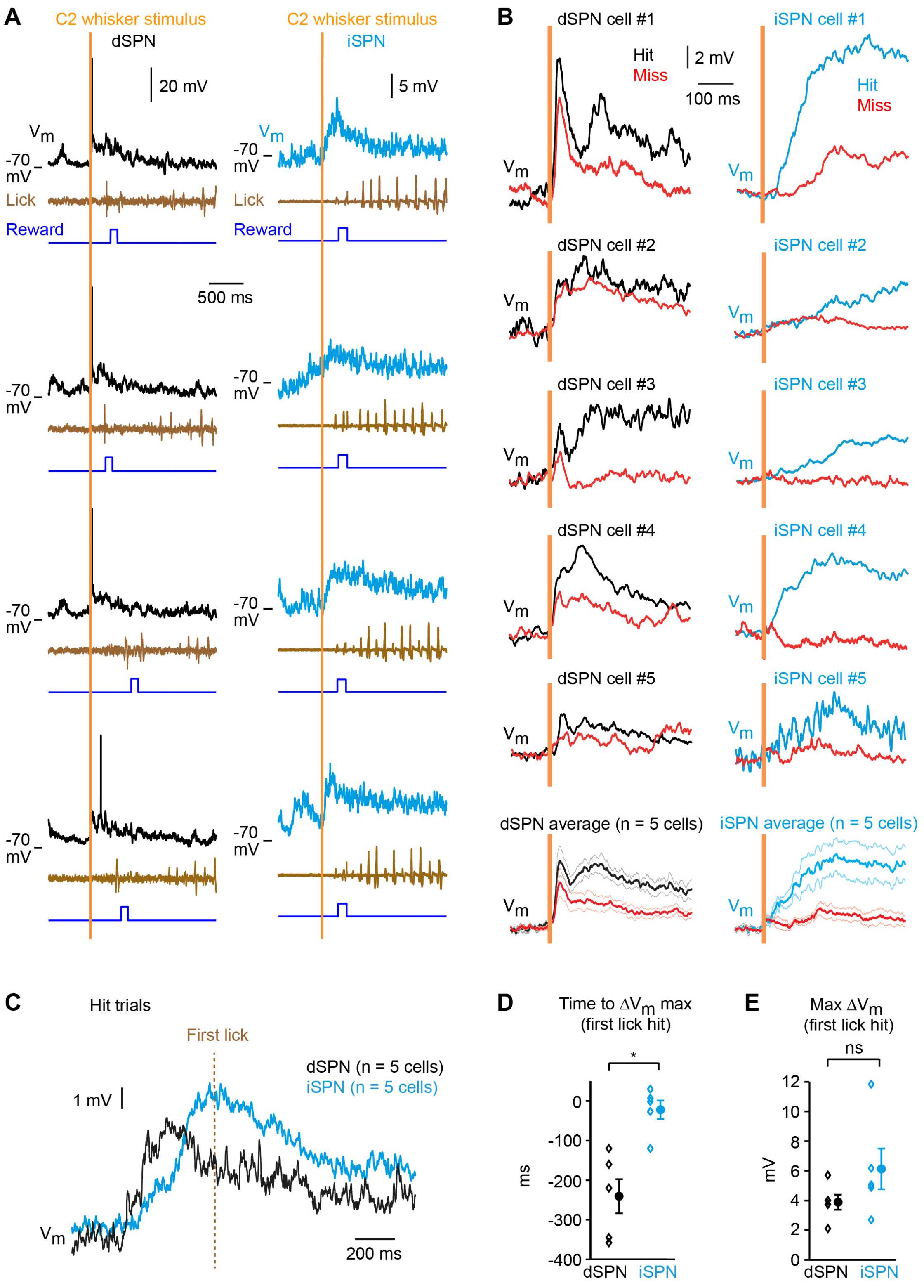

A
C2 whisker stimulus
dSPN
20 mV
Vm
-70 mV
Lick
Reward
C2 whisker stimulus
iSPN
5 mV
500 ms
B
dSPN cell #1
Hit
Miss
2 mV
100 ms
iSPN cell #1
dSPN cell #2
iSPN cell #2
dSPN cell #3
iSPN cell #3
dSPN cell #4
iSPN cell #4
dSPN cell #5
iSPN cell #5
dSPN average (n = 5 cells)
iSPN average (n = 5 cells)
C
Hit trials
First lick
dSPN (n = 5 cells)
iSPN (n = 5 cells)
1 mV
200 ms
D
Time to ΔVm max (first lick hit)
*
ms
0
-100
-200
-300
-400
dSPN
iSPN
E
Max ΔVm (first lick hit)
ns
mV
12
10
8
6
4
2
0

Figure S3. Membrane potential of dSPNs and iSPNs, related to Figure 3.

(A) Example V_m traces of 4 individual hit trials (black, left) from the dSPN shown in Figure 3A, showing strong early responses characteristic of this cell-type. Example V_m traces of 4 individual hit trials (blue, right) from the iSPN shown in Figure 3B.

(B) The average hit and miss trial V_m traces for each of the identified dSPNs (hit black, miss red, left) and iSPNs (hit blue, miss red, right). The grand average V_m traces (mean thick traces, SEM lighter traces) across hit and miss trials for the dSPNs (n = 5 cells, hit black, miss red, left) and iSPNs (n = 5 cells, hit blue, miss red, right).

(C) Grand average V_m traces for dSPNs (black, n = 5 cells) and iSPNs (blue, n = 5 cells) showing first lick triggered averages on hit trials. The V_m of dSPNs depolarized earlier than iSPNs before the first lick of hit trials.

(D) The time of the peak V_m depolarization on hit trials around the time of the first lick (t = 0 ms) occurred significantly earlier for dSPNs *vs* iSPNs (Wilcoxon-Mann-Whitney two-sample rank test p = 0.02).

(E) Peak V_m depolarization of traces aligned to the time of the first lick during hit trials compared to baseline V_m was not significantly different between dSPNs and iSPNs (Wilcoxon-Mann-Whitney two-sample rank test p = 0.2).

Values are mean ± SEM. *, $p < 0.05$. ns = non-significant.

Figure S4

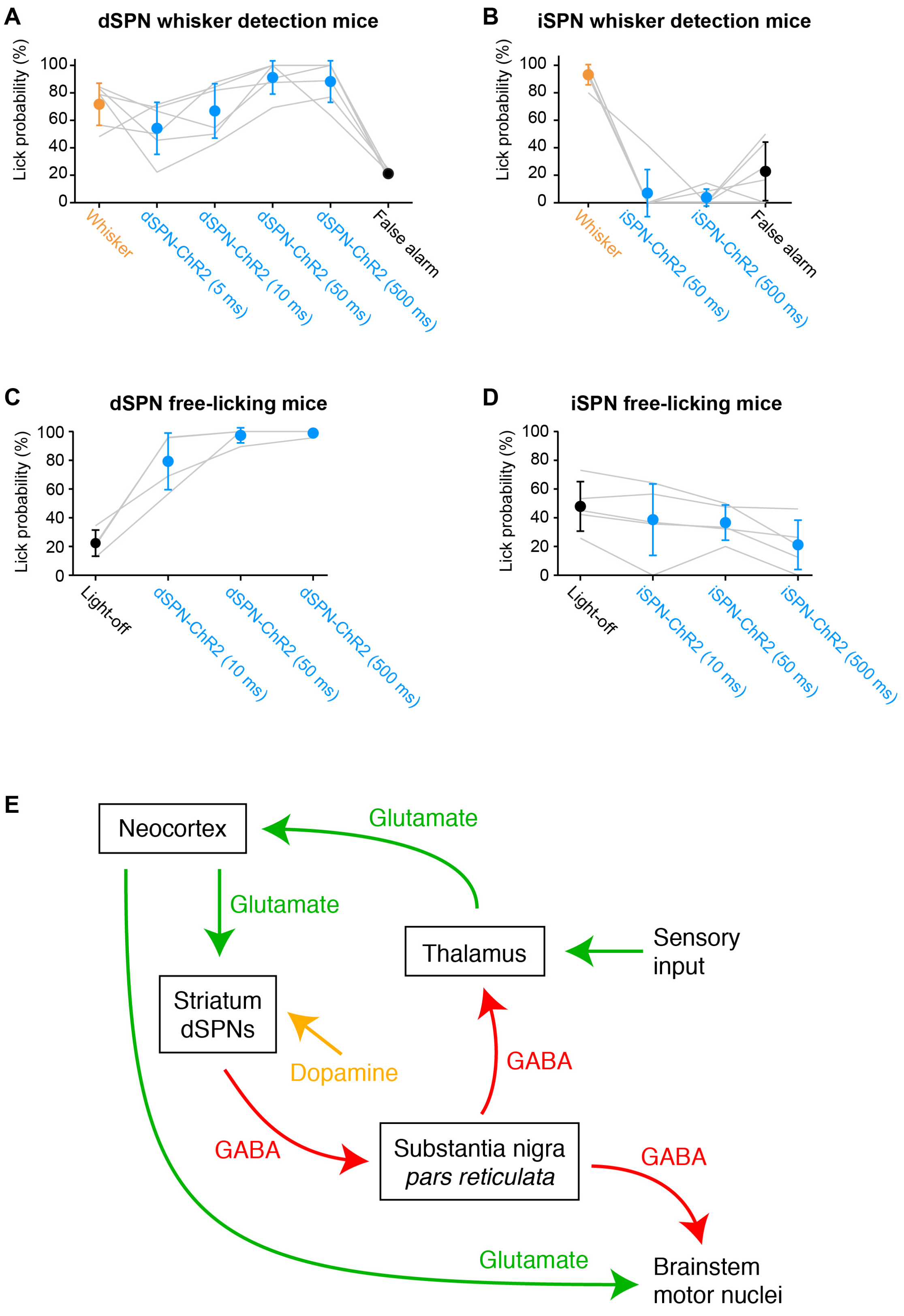
A
dSPN whisker detection mice
Lick probability (%)
100
80
60
40
20
0
Whisker
dSPN-ChR2 (5 ms)
dSPN-ChR2 (10 ms)
dSPN-ChR2 (50 ms)
dSPN-ChR2 (500 ms)
False alarm
B
iSPN whisker detection mice
Lick probability (%)
100
80
60
40
20
0
Whisker
iSPN-ChR2 (50 ms)
iSPN-ChR2 (500 ms)
False alarm
C
dSPN free-licking mice
Lick probability (%)
100
80
60
40
20
0
Light-off
dSPN-ChR2 (10 ms)
dSPN-ChR2 (50 ms)
dSPN-ChR2 (500 ms)
D
iSPN free-licking mice
Lick probability (%)
100
80
60
40
20
0
Light-off
iSPN-ChR2 (10 ms)
iSPN-ChR2 (50 ms)
iSPN-ChR2 (500 ms)
E
Neocortex
Glutamate
Glutamate
Thalamus
Sensory input
Striatum dSPNs
Dopamine
GABA
GABA
Substantia nigra pars reticulata
GABA
Glutamate
Brainstem motor nuclei

Figure S4. Optogenetic stimulation of dSPNs, but not iSPNs, readily substituted for whisker stimulation, related to Figure 4.

(A) The probability that a mouse trained in the whisker detection task will lick the reward spout (lick probability) is plotted *vs* various stimulation parameters. For dSPN-ChR2 mice, blue light stimulation, ranging from 5-500 ms in duration could effectively substitute for whisker stimulation. Performance of the mice with even the shortest light pulse used, 5 ms, was not statistically significantly different from whisker stimulation (n = 6 mice, Kruskal-Wallis test followed by Student-Newman-Keuls test $p > 0.05$) and with longer duration (50 and 500 ms) mice performed even better than with whisker stimulation (n = 6 mice, Kruskal-Wallis test followed by Student-Newman-Keuls test $p < 0.01$). Values are mean ± SD.

(B) For iSPN-ChR2 mice trained in the whisker detection task, blue light stimulation of 50 ms or 500 ms duration could not substitute for whisker stimulation, and performance in response to light stimulation in these animals was not different from the false alarm rate (n = 6 mice, Kruskal-Wallis test followed by Student-Newman-Keuls test $p > 0.05$). Values are mean ± SD.

(C) In order to investigate a striatal motor signal in the absence of training in the whisker detection task, we trained thirsty mice to lick a reward spout, and water was delivered anytime the mouse licked after a 4 s period without any lick. Random unrewarded optogenetic stimulation of dSPNs readily evoked licking in these highly motivated free-licking mice. Licking probability was significantly higher following blue light pulses of any duration (10 ms, 79.3 ± 19.8 %; 50 ms, 97.4 ± 5.3 %; or 500 ms, 98.9 ± 2.2 %) than in the absence of light pulse (Light-off, 22.4 ± 9.1 %) (n = 4 mice, Kruskal-Wallis test followed by Student-Newman-Keuls test $p < 0.01$). Values are mean ± SD.

(D) Optogenetic stimulation of iSPNs in free-licking mice did not evoke licking. Licking probability was not significantly different following blue light pulses of any duration (10 ms, 38.7 ± 24.9 %; 50 ms, 36.6 ± 12.3 %; or 500 ms, 21.2 ± 17.1 %) than in the absence of light pulse (Light-off, 47.9 ± 17.3 %) (n = 5 mice, Kruskal-Wallis test, $p > 0.05$). Values are mean ± SD.

(E) Schematic diagram illustrating pathways likely to contribute to the goal-directed sensorimotor transformation underlying the whisker detection task. Ascending somatosensory glutamatergic input from the brainstem brings

whisker sensory information to the thalamus. Thalamocortical neurons signal information to the neocortex. In a previous study we found that primary somatosensory neocortex participates causally in performance of the detection task (Sachidhanandam et al., 2013). The neocortex innervates many brain regions involved in motor control, including a strong projection to the striatum. The data in this study are consistent with direct-pathway striatal projection neurons (dSPNs) sending a fast transient inhibitory signal to the substantia nigra *pars reticulata* in response to detected whisker stimuli. Transient inhibition of GABAergic neurons in substantia nigra *pars reticulata* could then mediate disinhibition of thalamus and brainstem motor nuclei. The resulting enhanced activity in thalamus and brainstem motor nuclei could contribute to driving the learned goal-directed sensorimotor transformation underlying the whisker detection task.

Supplemental Experimental Procedures

Animal preparation and surgery

All experiments were carried out with 5-9 week old male and female mice in accordance with the Swiss Federal Veterinary Office (authorization 1628.3). D1-Cre, A2A-Cre and D2-Cre bacterial artificial chromosome (BAC) transgenic mice were obtained from Gene Expression Nervous System Atlas (GENSAT; founder line EY262 and EY217 for D1-Cre, KG139 for A2A-Cre and ER44 for D2-Cre), and purchased through the Mutant Mouse Regional Resource Centers (MMRRC). These mice were then crossed with lox-stop-lox (LSL) tdTomato reporter mice (B6.Cg-Gt(ROSA)26Sor$^{tm9(CAG-tdTomato)Hze/}$J) to obtain D1-Cre x LSL-tdTomato, A2A-Cre x LSL-tdTomato or D2-Cre x LSL-tdTomato mice that were implanted with a light-weight metal head-post and a recording chamber under ketamine/xylazine anesthesia. Three to seven days after surgery, all whiskers were trimmed on both sides except the C2 whiskers. Intrinsic signal optical imaging was carried out to locate the C2 barrel column in the left hemisphere.

Behavioral Training

One week after implantation, all whiskers were again trimmed except the C2 whisker on either side. Over a period of 1-2 days mice were adapted to head restraint. They were subsequently water deprived for 24 hours before behavioral training. During training in the detection task, mice received water exclusively in the behavioral setup, and were allowed brief free access (15 minutes) to wet food immediately thereafter in an individual cage. Iron filings were applied to the right C2 whisker at the beginning of each training session, allowing the whisker to be vertically deflected by a 1 ms current pulse passed through an electromagnetic coil placed immediately beneath the head of the mouse. Mice were then trained to associate this deflection of the C2 whisker with the availability of water at a reward spout placed within reach of their tongue. If the mouse licked the spout within the reward window (1 s), it was considered a hit trial, and the mouse received a drop of water. If not, it was considered a miss trial and no reward was delivered. Whisker stimuli were delivered without any preceding cues at random time intervals ranging

between 6 and 10 seconds. To discourage spontaneous licking, a 4 s timeout period was imposed during which no lick should occur in order to start a trial. Trials with whisker stimuli were randomly interleaved with ‘catch trials’ in which no stimulus was given. If licks occured during the response window of a catch trial it was considered a false alarm.

Mice were able to achieve a stable level of performance over the course of a few days (Figure S1), with a high hit rate and a low false alarm rate. Ambient white noise was played at all times to mask any potential auditory cues arising from whisker stimulation. Licks were detected with piezo-film attached to the reward spout. Behavioral control and data collection were carried out with custom written computer programs using either an ITC18 (Instrutech) interfaced through IgorPro (Wavemetrics) or a National Instruments board interfaced through LabView. Once the mice achieved a consistent level of performance (hit rate greater than 80% and false alarm rate less than 35%) they were considered adequately trained and were subsequently used for electrophysiological recordings or optogenetic manipulations.

The state of motivation of the mice plays an important role in determining the probability of licking in response to whisker stimulation. However, within the relatively short V_m recording periods we did not find any change in behavioral performance. The ‘miss rate’ of 49.7 ± 5.7 % (i.e. ‘hit rate’ of 50.3 ± 5.7 %) at the beginning of the recordings (first quarter of trials) did not differ appreciably from the ‘miss rate’ of 40.7 ± 6.3 % (i.e. ‘hit rate’ of 59.3 ± 6.3 %) at the end of the recordings (last quarter of the trials).

Electrophysiology

On the day of recording, a small (less than 1 mm diameter) craniotomy was made under isoflurane anesthesia over the dorsolateral striatum (stereotaxic coordinates: 0 mm anterior and 2.8-3.0 mm lateral of bregma). Mice were allowed to recover from anesthesia for two to four hours. Then, whole-cell patch-clamp recordings were obtained as previously described (Sachidhanandam et al., 2013; Reig and Silberberg, 2014). 6-8 MΩ glass pipettes were filled with a solution containing (in mM): 135 potassium gluconate, 4 KCl, 10 HEPES, 10 sodium phosphocreatine, 4 MgATP, 0.3

Na_3GTP (adjusted to pH 7.3 with KOH), to which 2-4 mg/ml biocytin was added. In some experiments, local field potential (LFP) was recorded with a 2-4 MΩ glass pipette filled with Ringer solution and lowered to a depth of 150-250 μm from the pia in the C2 barrel column. V_m and LFP signals were amplified using a Multiclamp 700B amplifier (Axon Instruments), Bessel filtered at 10 kHz, and digitized at 20 kHz by an ITC-18 (Instrutech Corporation) under the control of IgorPro (Wavemetrics). All patch-clamp recordings were obtained in current-clamp mode without injection of any current, except during the characterization of intrinsic electrophysiological properties. V_m was not corrected for liquid junction potentials.

At the start of each recording, a series of increasing current steps was injected into each neuron. We proceeded with the recording if the neuron displayed both a stable resting V_m and overshooting action potentials. The series resistance (also termed access resistance) of the recordings ranged from 25-40 MΩ. On average across recordings, our measurements of V_m in SPNs during task performance included 19.1 ± 2.1 hit trials and 12.6 ± 1.8 miss trials.

Optogenetic activation

D1-Cre (strain EY217) mice and A2A-Cre mice (4 week old male and females) were injected under isoflurane anesthesia with an adenoassociated virus (AAV) serotype 5 expressing double floxed inverted reading frame humanized ChR2 (H134R) fused to YFP under control of EF1α promoter (AAV2/5 DIO-EF1α-hChR2(H134R)-eYFP; virus made by Penn Vector Core). Prior to virus injection a ~0.5 mm craniotomy was made over the area of dorsolateral striatum (stereotaxic coordinates: 0 mm anterior and 2.6 mm lateral to bregma). A glass injection pipette was tip filled with the virus solution and lowered into the dorsolateral striatum. 200 nl of the virus solution was slowly injected at a depth of 2500 μm below the pia with a flow rate of 40-50 nl/min. The micropipette was left in position for 8-10 minutes and then slowly retracted to prevent backflow of virus along the shaft.

Mice expressing ChR2 were trained in the whisker detection task as described above, with the addition of ambient blue light. On the transfer test day, a craniotomy of ~1 mm diameter was opened over the dorsolateral

striatum, and animals were allowed to recover for at least 2 hours. A multimode fiber optic cable (Thorlabs; 300 μm) coupled to a 473 nm blue laser (GMP) was then lowered to a depth of ~2 mm into the brain directly above dorsolateral striatum. During behavioral testing, a third kind of trial (ChR2 stimulus trials) was randomly interleaved with whisker stimulus and catch trials. The light stimulus consisted of a single pulse of blue light of either 5, 10, 50 or 500 ms. At the end of the experiment, the mice were anesthetized and perfused so their brains could be recovered to verify both the injection sites and placement of the optic fiber.

We also carried out optogenetic stimulation experiments in free-licking mice, which were not trained in the whisker detection task, but were water-deprived and trained to lick the reward spout in order to obtain water rewards (Figure S4). Three weeks following virus injection, mice were water deprived and trained in a free-licking task where water was delivered any time the mouse would lick after a 4 s period of no licking. Mice were trained for 2-4 sessions. On the test day, an optical fiber was lowered to a depth of ~2 mm into the brain directly above dorsolateral striatum. During behavioral testing, we randomly applied light stimuli of 10, 50 or 500 ms, or no light pulse (Light-off). Licking in response to optogenetic stimuli was not reinforced by water rewards. Lick probability was computed within a 1 s time window following the onset of the optogenetic stimulus.

Histology and immunohistochemistry

After recording, the mice were perfused with a 4% paraformaldehyde (PFA) in 0.1 M PBS. Brains were post-fixed for maximum 24 hours in the same solution, which was then replaced by a 0.1 M PBS solution. 100 μm-thick coronal sections were cut using a semi-automated vibratome (VT1000S, Leica). Streptavidin coupled to Alexa 647 (1:2000, Invitrogen) was used to reveal biocytin filling of postsynaptic neurons. Images were obtained with a laser scanning confocal microscope (LSM 700, Zeiss) equipped with an oil-immersion 63x/1.4NA objective. Three-dimensional anatomical reconstructions were traced from confocal fluorescence image stacks using Neurolucida (MBF Bioscience).

Data analysis

All data analysis was performed in MATLAB using custom written algorithms. To assess the amplitude of the early sensory response, the maximum depolarization of V_m over 0-50 ms post stimulation was calculated and subtracted from the baseline (calculated to be the average V_m over the 500 ms before the stimulus). To measure the amplitude of the late depolarization, V_m was averaged across 50-250 ms post stimulus and the value subtracted from the baseline (the average V_m over the 500 ms before the stimulus). Fast Fourier Transforms (FFTs) were calculated for 2 second segments immediately prior to each whisker stimulus or catch trial. The amplitude of low frequency V_m fluctuations was calculated by integrating the calculated FFTs from 1-5 Hz. We used the first and second derivative of the membrane potential to calculate response onset. Slopes were obtained as $\Delta V/\Delta t$ between onset and $\Delta Vmax$ time interval.

The effects of spontaneous unrewarded licking upon striatal V_m was quantified across licking bouts that occurred at least 4 seconds prior to, and least 4 seconds after any whisker stimulation. In addition each spontaneous licking bout was separated by at least 2 seconds from the previous licking bout. The first lick in the licking bout was identified, and used to align epochs before averaging.

All values are presented as mean ± SEM (except Figures 4D and S4A-D, which show mean ± SD). Non-parametric statistical tests were used to assess significant differences. The Wilcoxon-Mann-Whitney two-sample rank test was used for unpaired samples (dSPNs *vs* iSPNs). The Wilcoxon signed rank test was used for paired samples (hit *vs* miss trials). For multiple comparisons, we first performed a Kruskal-Wallis test, then a nonparametric variation on the Student-Newman-Keuls test to compare between samples.
