# Supplementary material for: Cell-Type-Specific Sensorimotor Processing in Striatal Projection Neurons during Goal-Directed Behavior
Source: Neuron. 2015 Oct 21;88(2):298–305. doi: 10.1016/j.neuron.2015.08.039 (PMC4622932; doi:10.1016/j.neuron.2015.08.039)
Supplement: Document S2. Article plus Supplemental Information [file mmc2.pdf]

Report

Neuron

Cell-Type-Specific Sensorimotor Processing in Striatal Projection Neurons during Goal-Directed Behavior

Highlights

- Membrane potential recordings of striatal projection neurons in behaving mice
- Detected whisker stimuli evoke large sensorimotor responses in dorsolateral striatum
- Early sensory-evoked depolarization in striatonigral but not striatopallidal neurons
- Optogenetic substitution by striatonigral but not striatopallidal neurons

Authors

Tanya Sippy, Damien Lapray, Sylvain Crochet, Carl C.H. Petersen

Correspondence

carl.petersen@epfl.ch

In Brief

Sippy et al. measure cell-type-specific membrane potential dynamics in the striatum of head-restrained mice performing a simple goal-directed sensorimotor transformation. Their results are consistent with direct pathway striatonigral neurons delivering a fast "go" signal for action initiation.

CrossMark

Sippy et al., 2015, Neuron *88*, 298–305
October 21, 2015 ©2015 The Authors
http://dx.doi.org/10.1016/j.neuron.2015.08.039

CellPress

Cell-Type-Specific Sensorimotor Processing in Striatal Projection Neurons during Goal-Directed Behavior

Tanya Sippy,[1,2] Damien Lapray,[1] Sylvain Crochet,[1] and Carl C.H. Petersen[1,*]
[1]Laboratory of Sensory Processing, Brain Mind Institute, Faculty of Life Sciences, École Polytechnique Fédérale de Lausanne (EPFL), Lausanne CH-1015, Switzerland
[2]Present address: Department of Psychiatry, New York University Langone Medical Center, NY 10016, USA
*Correspondence: carl.petersen@epfl.ch
http://dx.doi.org/10.1016/j.neuron.2015.08.039
This is an open access article under the CC BY license (http://creativecommons.org/licenses/by/4.0/).

SUMMARY

Goal-directed sensorimotor transformation drives important aspects of mammalian behavior. The striatum is thought to play a key role in reward-based learning and action selection, receiving glutamatergic sensorimotor signals and dopaminergic reward signals. Here, we obtain whole-cell membrane potential recordings from the dorsolateral striatum of mice trained to lick a reward spout after a whisker deflection. Striatal projection neurons showed strong task-related modulation, with more depolarization and action potential firing on hit trials compared to misses. Direct pathway striatonigral neurons, but not indirect pathway striatopallidal neurons, exhibited a prominent early sensory response. Optogenetic stimulation of direct pathway striatonigral neurons, but not indirect pathway striatopallidal neurons, readily substituted for whisker stimulation evoking a licking response. Our data are consistent with direct pathway striatonigral neurons contributing a "go" signal for goal-directed sensorimotor transformation leading to action initiation.

INTRODUCTION

A key function of the brain is to interpret incoming sensory information in the context of learned associations in order to guide adaptive behavior. However, the precise neuronal circuits and causal mechanisms underlying goal-directed sensorimotor transformations remain to be clearly defined for the mammalian brain. The basal ganglia are thought to be involved in action initiation and selection (Alexander and Crutcher, 1990; Graybiel et al., 1994; Grillner et al., 2005; Jin and Costa, 2010; Stephenson-Jones et al., 2011), and their dysfunction is associated with sensorimotor disorders, including Parkinson's disease (Albin et al., 1989; DeLong, 1990; Kravitz et al., 2010). The input layer of the basal ganglia, the striatum, receives glutamatergic inputs from various cortical regions and the thalamus, as well as a significant dopaminergic projection, making this structure well-suited for integration of sensory input with reward signaling to produce appropriate motor output.

The vast majority of neurons in the striatum are GABAergic striatal projection neurons (SPNs). The SPNs can be subdivided according to their distinct long-range axonal projection patterns that correlate with differential gene expression (Gerfen et al., 1990; Bateup et al., 2010; Gerfen et al., 2013). The direct-pathway striatonigral neurons (dSPNs) expressing D1 receptors project to the substantia nigra and are often considered to form part of a "go" signaling pathway for action initiation, whereas the indirect pathway striatopallidal neurons (iSPNs) expressing D2 and A2A receptors project to the external segment of the globus pallidus and are thought to participate in "no go" signals (Durieux et al., 2009; Kravitz et al., 2012; Tai et al., 2012; Freeze et al., 2013). However, recent studies have failed to detect differences in the activity patterns of dSPNs versus iSPNs during task performance (Cui et al., 2013), questioning the validity of "go" and "no go" roles for these pathways.

Here, we investigate the role of the striatum in a simple sensorimotor task in which mice learn to lick for water reward in response to a single brief whisker deflection (Sachidhanandam et al., 2013). Because SPNs in vivo characteristically have low action potential firing rates (Wilson and Groves, 1981; Reig and Silberberg, 2014), we used whole-cell recordings to study both subthreshold and suprathreshold membrane potential (V_m) activity of these neurons. Our recordings revealed strong task-related V_m dynamics in the dorsolateral striatum, with larger depolarizations on hit trials than miss trials. Interestingly, this activity differed substantially between the direct pathway striatonigral neurons and the iSPNs, with a fast transient excitation specifically in dSPNs. Optogenetic stimulation of dSPNs during task performance was consistent with brief excitation of the direct pathway playing a causal role in the sensorimotor transformation.

RESULTS

We trained head-restrained mice to perform a simple goal-directed sensorimotor transformation in order to correlate behavioral performance with neuronal activity in the dorsolateral striatum. In our task, we delivered single 1-ms-duration

deflections to the C2 whisker and trained mice to report detected stimuli by licking a reward spout (Figures 1A and S1) (Sachidhanandam et al., 2013). After mice were well-trained, we obtained whole-cell V_m recordings in the dorsolateral striatum during task performance (hit rate 60.9% ± 3.5%, false alarm rate 11.9% ± 2.8%, n = 30 cells) (Figure 1B). The dorsolateral striatum receives prominent excitatory glutamatergic input from primary somatosensory cortex (S1) (Figure 1C) (Wall et al., 2013; Reig and Silberberg, 2014), and S1 cortex is known to play a causal role in performance of this detection task (Sachidhanandam et al., 2013).

The whole-cell recording technique provides information about incoming subthreshold postsynaptic potentials and action potential output, which is particularly useful in brain regions dominated by neurons that have low firing rates. We recorded from 30 SPNs in 25 mice, and each neuron showed obvious task-related V_m dynamics. There was substantial diversity across different recordings, with many neurons (n = 24 cells) showing mostly subthreshold V_m changes (Figures 1D–1F) and a minority of neurons (n = 6 cells) firing task-related action potentials at high rates (Figures 1G–1I). Biocytin labeling introduced through the whole-cell recording pipette allowed anatomical identification of 27 out of 30 cells as SPNs, with their characteristic spiny morphology. The three other recorded neurons were also considered as SPNs, since they had similar electrophysiological properties to the identified cells, and these properties are not consistent with any other known striatal cell type.

V_m of SPNs during Task Performance

Important information can be learned about the neuronal activity underlying the conversion of sensory signals into goal-directed motor output by comparing hit and miss trials. In our recordings from SPNs in the dorsolateral striatum, we found striking differences in V_m dynamics depending upon behavioral outcome (hit versus miss), both for individual neurons (Figures 1D–1I) and analyzed across the population of recorded neurons (n = 30 cells) (Figure 2A). The grand average V_m showed two obvious phases, an early transient depolarization and a later longer-lasting depolarization (Figure 2A). The peak of the early (0–50 ms) depolarizing sensory response was significantly larger in hit trials (hit 3.0 ± 0.4 mV, miss 2.2 ± 0.4 mV, n = 30 cells, Wilcoxon signed rank test p = 0.002) (Figure 2B). During the secondary late phase (50–250 ms after whisker stimulus), the average evoked V_m depolarization in the dorsolateral striatum was also significantly larger in hit trials (hit 4.0 ± 0.5 mV, miss 1.3 ± 0.3 mV, n = 30 cells, Wilcoxon signed rank test p = 2 × 10^{-6}) (Figure 2C). Action potential firing was also increased in hit trials (hit 1.40 ± 0.68 Hz, miss 0.10 ± 0.05 Hz, n = 30 cells, Wilcoxon signed rank test p = 0.016) (Figure 2D).

We were curious if pre-stimulus differences in striatal V_m could account for behavioral performance. We therefore compared the pre-stimulus baseline V_m, the fast fourier transform of the V_m and the correlation of the V_m with the local field potential recorded in S1 in the 2 s preceding each whisker stimulus. However, similar to results in S1 (Sachidhanandam et al., 2013), we did not find differences in the pre-stimulus V_m in the striatum comparing hit and miss trials (Figure S2).

The striatum is thought to be important for initiation and control of movement, leading us to question how the V_m of SPNs was modulated by licking during the behavioral task. We therefore aligned the V_m traces of SPNs with respect to the mouse's first lick during both stimulus (hit) trials and unrewarded spontaneous licking (Figure 2E). The V_m was significantly depolarized from baseline with respect to licking during both hit trials and spontaneous licking (change in V_m from baseline on hit trials 4.8 ± 0.6 mV, n = 30 cells, Wilcoxon signed rank test p = 1.7 × 10^{-6}; change in V_m from baseline for spontaneous licking 3.0 ± 0.7 mV, n = 30 cells, Wilcoxon signed rank test p = 8.5 × 10^{-6}) (Figures 2E and 2F). The depolarization began hundreds of milliseconds before licking, and excitation of striatal neurons could therefore contribute to initiating the licking motor response.

V_m Dynamics of Direct and Indirect Pathway SPNs

In a subset of our experiments, we were able to unambiguously identify the type of SPN through post hoc histology. The intracellular pipette solution contained biocytin, allowing for colocalization of fluorescent biocytin staining with tdTomato fluorescence in D1-Cre mice (for dSPNs) and D2-Cre or A2A-Cre mice (for iSPNs) crossed with LSL-tdTomato reporter mice (Figures 3A and 3B) (Madisen et al., 2010; Gerfen et al., 2013). A neuron was identified as a dSPN or iSPN only if the biocytin-filled neuron also expressed tdTomato. Neurons that were filled with biocytin but did not express tdTomato were not included in the analysis in order to avoid incorporating false negatives into our dataset.

In this subset of positively defined subtypes of SPNs, it was apparent that the early (0–50 ms) response was much more pronounced in dSPNs compared to iSPNs (Figures 3C and S3). Quantification of the slope of the early sensory response in hit trials revealed it to be significantly faster in dSPNs (dSPN 0.32 ± 0.10 mV.ms^{-1}, n = 5 cells; iSPN 0.10 ± 0.02 mV/ms, n = 5 cells; Wilcoxon Mann-Whitney two-sample rank test p = 0.008) (Figure 3D). The amplitude of the early response in hit trials was also significantly larger in dSPNs when compared to iSPNs (dSPN 6.0 ± 1.4 mV, n = 5 cells; iSPN 2.6 ± 0.5 mV, n = 5 cells; Wilcoxon Mann-Whitney two-sample rank test p = 0.03) (Figure 3E).

We also compared the late phase (50–250 ms post-stimulus) in dSPNs and iSPNs. We found that during the late phase, the two neuron types were equally depolarized on hit trials (dSPNs 4.6 ± 0.6 mV, n = 5 cells; iSPNs 5.1 ± 1.4 mV, n = 5 cells; Wilcoxon Mann-Whitney two-sample rank test, p = 1) (Figure 3F). Our data therefore reveal a strong rapid transient sensory-evoked depolarization specifically in dSPNs, which could contribute a "go" signal to initiate licking behavior.

Optogenetic Activation of the Direct and Indirect Pathways

In order to test our hypothesis that the early response in dSPNs might contribute to initiate movement, we sought to activate this pathway specifically in the context of the detection task. Toward this goal, we made use of an optogenetic approach. We injected Cre-dependent adeno-associated viral vectors encoding channelrhodopsin-2 (ChR2) linked to YFP into the dorsolateral

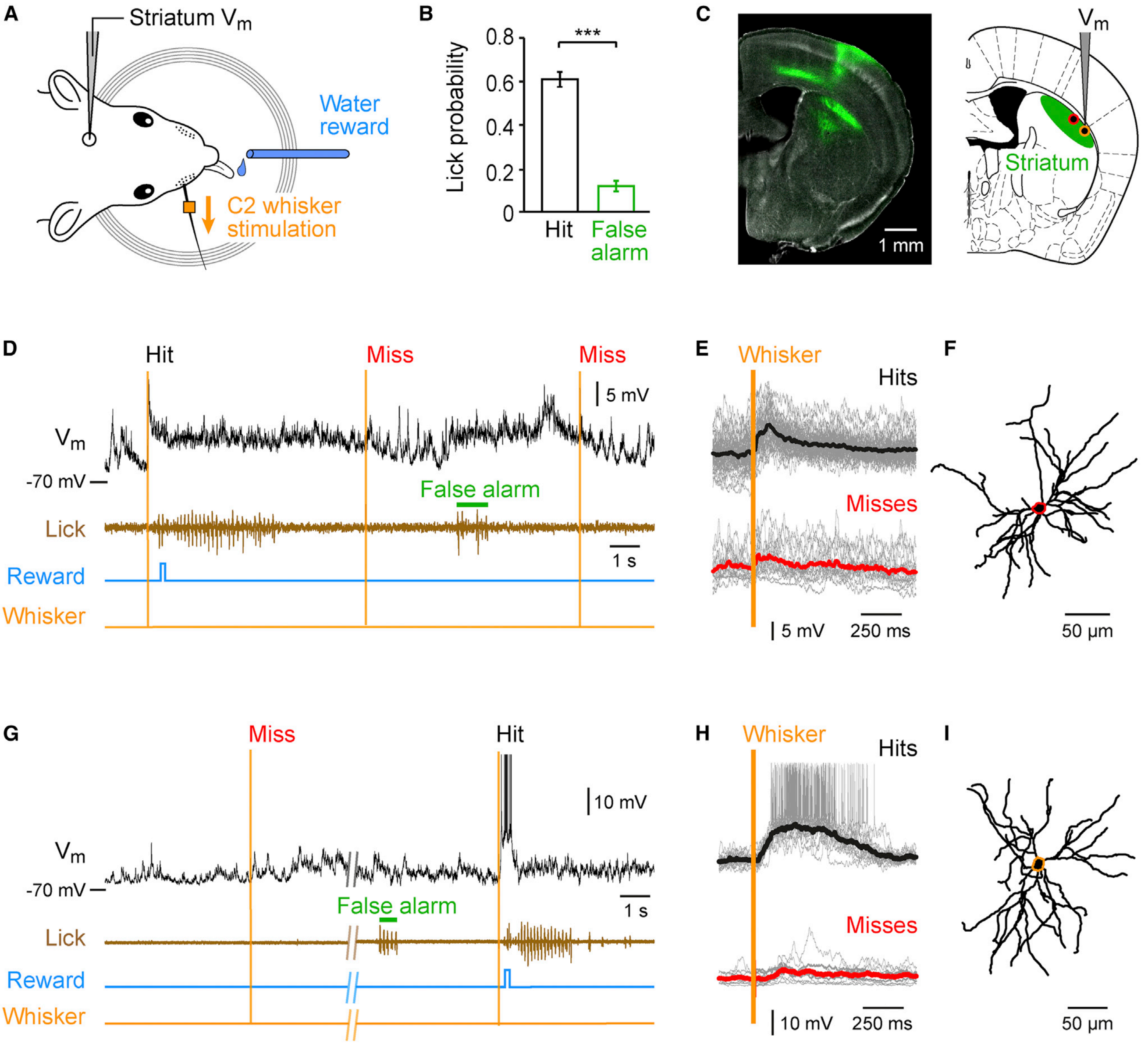

Figure 1. V_m Recordings from Identified Neurons in the Dorsolateral Striatum of Behaving Mice

(A) Mice were head-restrained above an electromagnetic coil. Metal particles were placed on the right C2 whisker, which was deflected with a 1 ms current pulse delivered to the electromagnetic coil at random time intervals (6–10 s) without any preceding cue. Mice learned to lick the spout within 1 s of the whisker stimulus in order to receive a water reward. To control for random licking, stimulation trials were interleaved with catch trials in which no stimulation was given.

(B) Mice learned the task over ~1 week of training, reaching stable performance with hit rates (black) significantly higher than false alarm rates (green). Data are shown for performance during the electrophysiological recordings (n = 30 cells recorded across 25 mice, $p = 1.7 \times 10^{-5}$).

(C) An anterograde tracer, AAV2-Synapsin-GFP, was injected into the left C2 barrel column showing prominent axonal innervation of left dorsolateral striatum (n = 3 mice) (left). Schematic coronal section showing the area of striatum (green) targeted for whole-cell recordings during the detection task (right). Color-coded circles show locations of the two example cells in (D)–(F) (red) and (G)–(I) (orange).

(D) Example trace showing subthreshold V_m activity in an SPN during the detection task. V_m (black) of the neuron was recorded simultaneously with measurement of licking (brown) from the piezo-film attached to the reward spout. Licking within the 1 s reward window after C2 whisker stimulus (orange) opened a valve to deliver water reward (blue).

(E) V_m of the SPN shown in (D), for all hit trials (black average, gray individual, n = 49 trials) and miss trials (red average, gray individual, n = 24 trials).

(F) Dendritic structure of the recorded neuron.

(G) V_m of a neuron with suprathreshold task-related activity.

(H) Average traces from this neuron showing all hit (n = 21) and miss (n = 10) trials.

(I) Dendritic structure of this neuron.

Values are mean ± SEM. ***$p < 0.001$. See also Figure S1.

CellPress

striatum of either D1-Cre or A2A-Cre mice, thereby expressing the opsin specifically in either dSPNs or iSPNs, respectively. Antibody staining of YFP revealed direct pathway dSPN axons projecting to substantia nigra pars reticulata (SNr) in D1-Cre mice (Figure 4A, and indirect-pathway iSPN axons innervating the external segment of globus pallidus (GPe) in A2A-Cre mice (Figure 4B). The mice were trained to detect whisker stimulation following our standard training procedures, except for the addition of blue background light. After stable whisker detection performance was reached, on a given transfer-test day, the first blue light flashes were introduced to the striatal neurons via an optical fiber. The optogenetic stimuli (ranging from 5–500 ms in duration) were randomly interleaved with standard whisker stimulation trials and catch trials without whisker stimulation (Figure S4). Brief, single optogenetic stimuli delivered to dSPNs readily substituted for whisker stimulation and evoked robust licking (Figure 4C). Across all mice tested, the hit rate evoked by 50-ms ChR2 stimulation of dSPNs (91% ± 5%, n = 6 mice) was significantly higher than the false alarm rate (25% ± 5%, n = 6 mice, Kruskal-Wallis test followed by Student-Newman-Keuls test $p < 0.005$), and not different from the whisker stimulus evoked hit rate (76% ± 8%, n = 6 mice, Kruskal-Wallis test followed by Student-Newman-Keuls test $p > 0.05$) (Figure 4D). In contrast, optogenetic stimulation of iSPNs did not induce licking (Figures 4D and S4). The hit rate evoked by 50-ms ChR2 stimulation of iSPNs (7% ± 7%, n = 6 mice) was significantly lower than the whisker-stimulus-evoked hit rate (93% ± 3%, n = 6 mice, Kruskal-Wallis test followed by Student-Newman-Keuls test $p < 0.005$) and not significantly different from the false alarm rate (16% ± 7%, n = 6 mice, Kruskal-Wallis test followed by Student-Newman-Keuls test, $p > 0.05$). Therefore, only activation of dSPNs (and not iSPNs) reliably substituted for sensory stimulation during the detection task.

We also carried out the same optogenetic stimulation experiments in free-licking thirsty mice, which were not trained in the whisker detection task but which were trained to lick the spout in order to obtain water reward. In these highly motivated mice, stimulation of dSPNs, but not iSPNs, evoked licking (Figure S4). This suggests that the licking evoked by dSPN stimulation in the mice performing the whisker detection task relates more to a motor signal than a sensory signal.

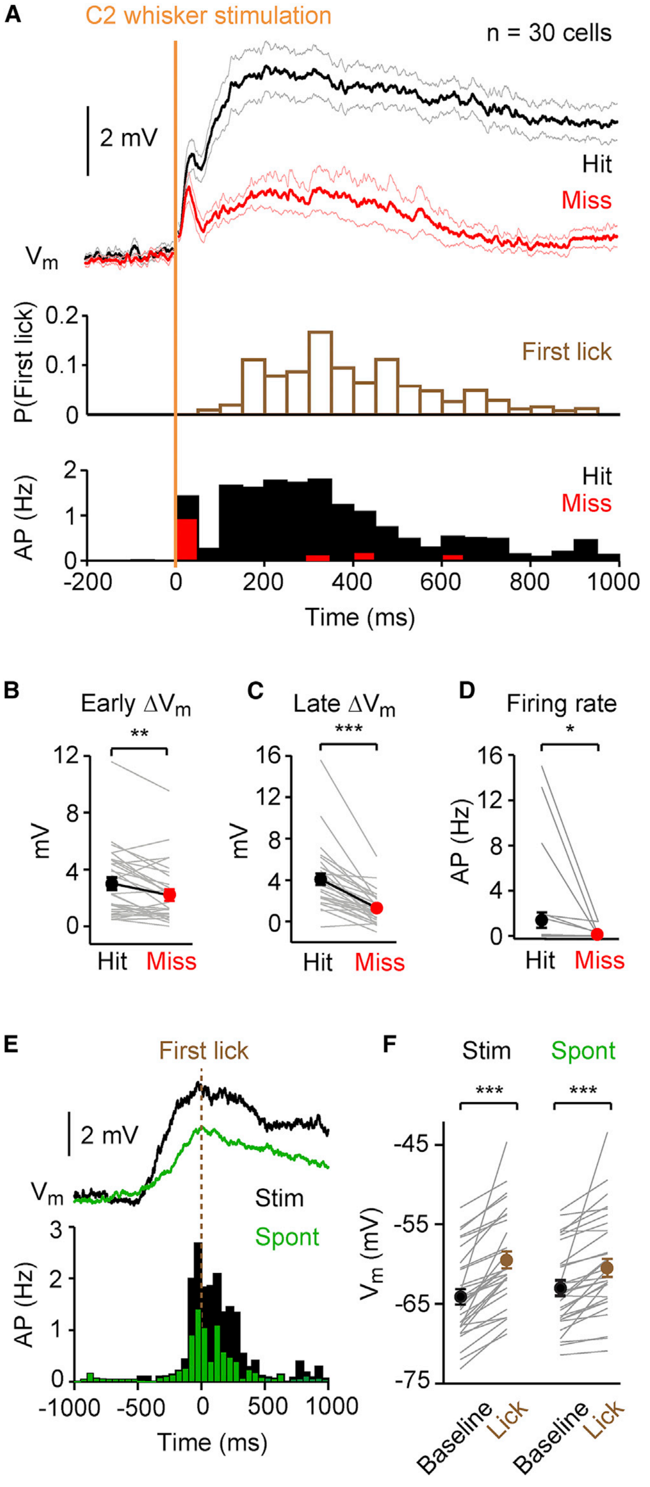

Figure 2. V_m of Striatal Neurons Correlates with Behavioral Performance

(A) Grand average V_m across all SPNs recorded in response to whisker stimulation during hit (black) and miss (red) trials (above). Lighter shaded lines represent SEM. The time of the first lick in response to whisker stimulation across all hit trials (middle). The PSTH of action potential firing during hit (black) and miss (red) trials (below).

(B) The early ΔV_m (calculated as the peak depolarization in the first 50 ms after whisker stimulation relative to prestimulus baseline V_m) was significantly larger during hit compared to miss trials (n = 30 cells, Wilcoxon signed rank test $p = 0.0024$).

(C) Late ΔV_m (calculated as the mean change in V_m from baseline to a period 50–250 ms after whisker stimulation) was significantly larger during hit trials versus miss trials (n = 30 cells, Wilcoxon signed rank test $p = 2.3 \times 10^{-6}$).

(D) The firing rate of SPNs was significantly higher during hit versus miss trials (n = 30 cells, Wilcoxon signed rank test $p = 0.02$).

(E) Average V_m and PSTH of SPNs around the time of the first lick on hit trials ("Stim," black) and unrewarded spontaneous licking ("Spont," green).

(F) The V_m depolarized significantly from baseline before the time of the first lick for both hits ("Stim," n = 30 cells, Wilcoxon signed rank test $p = 1.7 \times 10^{-6}$) and unrewarded spontaneous licking ("Spont," n = 30 cells, Wilcoxon signed rank test $p = 8.5 \times 10^{-6}$).

Values are mean ± SEM. $^*p < 0.05$; $^{**}p < 0.01$; $^{***}p < 0.001$. See also Figure S2.

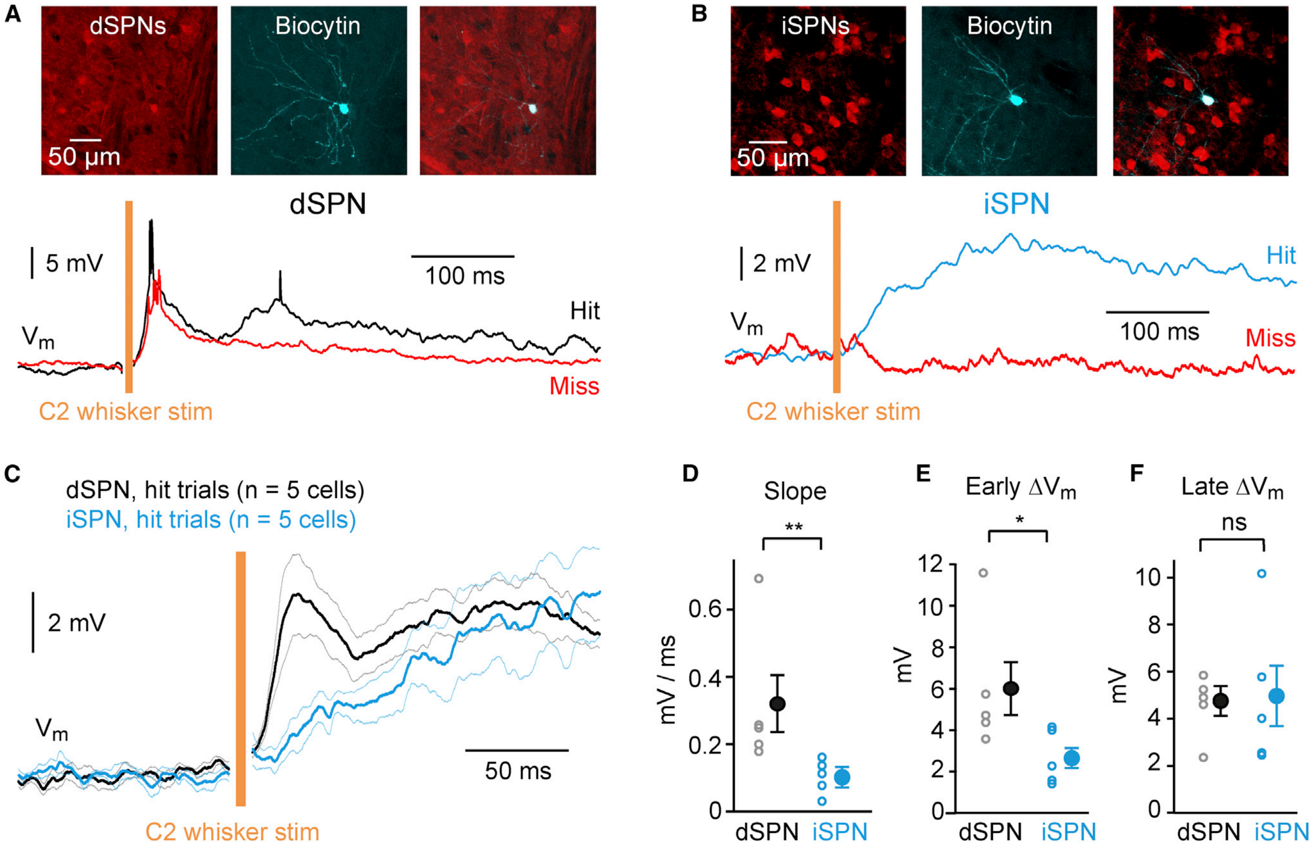

Figure 3. Cell-Type-Specific Sensorimotor Processing during the Detection Task

(A) A D1-Cre mouse was crossed with a LSL-tdTomato mouse, driving expression of tdTomato in dSPNs (upper left). The recorded neuron was filled with biocytin and stained with Alexa-647 (upper middle). The labeled neuron was considered as a dSPNs because the Alexa-647 signal co-localized with tdTomato (upper right). Average hit and miss traces from the example positively identified dSPN (below).

(B) iSPNs express tdTomato in an A2A-Cre × LSL-tdTomato mouse (upper left). The recorded neuron was filled with biocytin and stained with Alexa-647 (upper middle). The biocytin stain co-localized with tdTomato defining this neuron as an iSPN (upper right). Average hit and miss traces (below) from the example positively identified iSPN.

(C) Grand average V_m of hit trials across all positively identified dSPNs (black, n = 5 cells) and iSPNs (blue, n = 5 cells), showing an early sensory response specifically in dSPNs. Lighter shaded lines represent SEM.

(D) The slope of the early response was significantly larger in dSPNs versus iSPNs (n = 5 of each cell type, Wilcoxon Mann-Whitney two-sample rank test, $p = 0.008$).

(E) The amplitude of the early response (Early ΔV_m), was significantly larger for dSPNs versus iSPNs (n = 5 of each cell type, Wilcoxon Mann-Whitney two-sample rank test, $p = 0.03$).

(F) The late response (Late ΔV_m) was not significantly different in dSPNs versus iSPNs (n = 5 of each cell type, Wilcoxon Mann-Whitney two-sample rank test, $p = 1$).

Values are mean ± SEM. *$p < 0.05$; **$p < 0.01$; ns, non-significant. See also Figure S3.

DISCUSSION

By using the whole-cell recording technique, we characterized the V_m of dorsolateral SPNs in awake, behaving mice. We examined changes in subthreshold and suprathreshold activity during a sensorimotor task requiring motivation and found that the V_m of SPNs strongly correlates with behavioral performance. Only neurons of the direct striatonigral pathway exhibited a prominent early sensory response, and only optogenetic stimulation of the direct striatonigral pathway substituted for peripheral stimulation. Our results extend current knowledge of V_m dynamics of SPNs and lend support for a mechanism by which the direct pathway striatonigral neurons contribute to initiate movement in the context of motivation.

V_m Measurements in Striatum and Cortex during the Detection Task

We previously measured V_m in the primary somatosensory cortex (S1) during the same detection task as used in this study (Sachidhanandam et al., 2013). It is therefore interesting to consider the similarities and differences between these closely related brain areas in their V_m dynamics evoked by the 1-ms deflection of the C2 whisker during task performance. In both cortex and striatum, we found biphasic depolarizing V_m

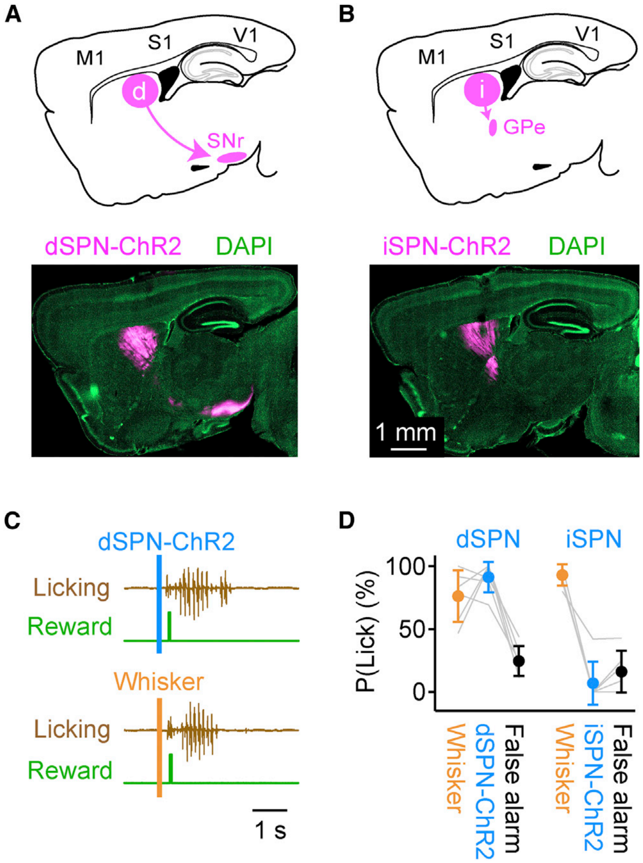

Figure 4. Optogenetic Stimulation of dSPNs, but Not iSPNs, Substitutes for Whisker Stimulation during the Detection Task

(A) Schematic sagittal section showing the direct-pathway projection (dSPNs) from striatum to SNr (above). Fluorescence image of a sagittal section from a D1-Cre mouse injected in the dorsolateral striatum with AAV-DIO-ChR2-YFP (below). Antibody staining for YFP (magenta) in axons shows the projection of dSPNs to SNr. DAPI staining of cell nuclei shown in green.

(B) Schematic of sagittal section showing indirect-pathway (iSPNs) projecting from striatum to the external segment of the globus pallidus (GPe). An A2A-Cre mouse was infected with AAV-DIO-ChR2-YFP in the dorsolateral striatum and antibody staining for YFP (magenta) shows the iSPN projection to GPe.

(C) On the transfer test day, 50-ms flashes of blue light delivered into the dorsolateral striatum (above) were interleaved with whisker stimuli (below) and trials without stimulation. As shown in these example traces, blue light activation of dSPNs was able to drive licking.

(D) Performance of dSPN-ChR2 mice in response to a 50-ms blue light stimulus on the transfer test day was similar to whisker stimulus trials (n = 6 mice, Kruskal-Wallis test followed by Student-Newman-Keuls test, $p > 0.05$) and significantly above the false alarm rate (n = 6 mice, Kruskal-Wallis test followed by Student-Newman-Keuls test, $p < 0.005$). However, performance of iSPN-ChR2 mice with 50-ms blue light was significantly lower than for whisker stimuli (n = 6 mice, Kruskal-Wallis test followed by Student-Newman-Keuls test, $p < 0.005$), and similar to the false alarm rate in these animals (n = 6 mice, Kruskal-Wallis test followed by Student-Newman-Keuls test, $p > 0.05$).

Values are mean ± SD. See also Figure S4.

responses consisting of an early sensory component and a later motor-related component. However, there were important qualitative and quantitative differences between the responses in cortex and striatum.

In layer 2/3 of S1 cortex, we found that the early sensory response is present in all neurons and does not differ between hit and miss trials (Sachidhanandam et al., 2013). In striatum, however, the early sensory response was specifically found in dSPNs (Figure 3) and was significantly larger in amplitude during hit trials (Figure 2). dSPNs in dorsolateral striatum receive strong input from whisker S1 (Wall et al., 2013; Reig and Silberberg, 2014), and corticostriatal input is likely to contribute importantly to the early sensory response in dSPNs. In future studies, it will therefore be important to determine if corticostriatal projection neurons in S1 provide differential excitation to SPNs on hit versus miss trials or whether this is a result of synaptic computation within the striatum or other neural circuit.

The late depolarization was larger in hit trials compared to miss trials both in striatum (Figure 2) and in layer 2/3 of S1 cortex (Sachidhanandam et al., 2013). However, the difference between the late depolarization in hits and misses is much larger in the dorsolateral SPNs compared to S1 cortex. The late depolarization (50–250 ms) follows the early sensory response but precedes licking motor output. Given that striatal V_m depolarized before both rewarded and spontaneous licking (Figure 2), the enhanced late phase in striatum on hit trials might, at least in part, be a motor-related signal, perhaps selectively promoting licking while inhibiting other motor output. Interestingly, the late depolarization was equally large in dSPNs and iSPNs, unlike the early sensory response (Figure 3). Whereas dSPNs receive stronger input from sensory cortex, iSPNs receive stronger input from motor cortex (Wall et al., 2013), and in future work, it will be interesting to investigate the differential cortical inputs to dSPNs and iSPNs during the early and late responses of hit trials.

The Early Response as a "Go" Signal in Direct Pathway Striatal Neurons

We have shown that a brief excitatory signal in dSPNs correlates with (Figures 1, 2, and 3) and is sufficient for (Figure 4) task performance. We speculate that such a signal could arise from reward-feedback during task learning through dopamine-related synaptic plasticity at corticostriatal synapses, with D1 receptor signaling helping potentiate synaptic input from S1 onto dSPNs and D2 receptor signaling perhaps promoting synaptic depression in iSPNs (Kreitzer and Malenka, 2008; Gerfen and Surmeier, 2011; Tritsch and Sabatini, 2012; Kress et al., 2013; Shepherd, 2013; Shan et al., 2014). Transient excitation of dSPNs could contribute to initiating licking motor output through at least two different circuits downstream of the SNr (Figure S4). Increased dSPN firing will inhibit the tonically active GABAergic SNr neurons projecting to downstream brainstem motor regions, thus causing disinhibition and enhancing motor output (Grillner et al., 2005; Freeze et al., 2013). GABAergic neurons in the SNr also project to the thalamus, which therefore becomes disinhibited by the dSPN "go" signal. Increased thalamic activity could contribute to late cortical depolarization, known to correlate with and contribute to task performance (Sachidhanandam et al., 2013), which could then form part of a recurrent, positive feedback loop through striatal-thalamo-cortical circuits.

Beyond the initial 50-ms-duration ‘‘go’’ excitation signal in dSPNs, the two types of SPNs behaved in a very similar way, with both cell types depolarizing equally at late times during hit trials (Figure 3E). It may thus be the fast signaling of the whisker detection task that gives our experimental paradigm sufficient temporal precision to uncover the specific dSPN ‘‘go’’ signal, which might not have been resolved in previous measurements (Cui et al., 2013). It should be noted, however, that due to the sparse AP firing rates of SPNs, our study includes only a small number of SPNs with appreciable spiking, and further studies will therefore be important to better characterize cell-type-specific firing patterns of striatal neurons during diverse behaviors.

Our data are consistent with corticostriatal signals contributing to simple goal-directed sensorimotor transformation (Znamenskiy and Zador, 2013; Xiong et al., 2015), perhaps resulting from learning under guidance of dopamine signals evoked by whisker stimulation serving as a reward predictor (Schultz et al., 1997; Schultz, 1998; Cohen et al., 2012). To test these hypotheses, in future experiments it will be important to record and manipulate the activity of dopaminergic neurons and also to test whether corticostriatal input from the C2 barrel column in S1 undergoes learning-induced plasticity that is necessary and sufficient for task performance.

EXPERIMENTAL PROCEDURES

Animal Preparation and Surgery

All experiments were carried out in accordance with protocols approved by the Swiss Federal Veterinary Office. For electrophysiological recordings, D1R-Cre, D2R-Cre, and A2AR-Cre mice (Gerfen et al., 2013) were crossed with Lox-STOP-Lox-tdTomato (LSL-tdTomato) reporter mice (Madisen et al., 2010). For optogenetic experiments, AAV-DIO-ChR2 virus was stereotactically injected into D1R-Cre or A2AR-Cre mice. A metal head-holder was implanted under anesthesia.

Behavioral Training

Mice were trained to lick a water-reward spout in response to single 1-ms-duration deflections of the C2 whisker following previously described procedures (Sachidhanandam et al., 2013).

Electrophysiology

Whole-cell patch-clamp recording electrodes (5–7 MΩ) were filled with an intracellular solution containing (in mM) 135 potassium gluconate, 4 KCl, 10 HEPES, 10 sodium phosphocreatine, 4 MgATP, and 0.3 Na_3GTP (adjusted to pH 7.3 with KOH), to which 3 mg/ml biocytin was added. V_m was recorded using a Multiclamp 700B amplifier without injection of holding current and was not corrected for liquid junction potentials.

Optogenetics

Blue light was delivered using a 300-μm-diameter optical fiber coupled to a 473 nm laser inserted into the brain directly above dorsolateral striatum.

Data Analysis

Electrophysiological and behavioral data were analyzed in Matlab. Data are presented as mean ± SEM throughout the text and figures, except Figure 4D, which shows mean ± SD.

SUPPLEMENTAL INFORMATION

Supplemental Information includes four figures and Supplemental Experimental Procedures and can be found with this article online at http://dx.doi.org/10.1016/j.neuron.2015.08.039.

AUTHOR CONTRIBUTIONS

T.S., D.L., S.C., and C.C.H.P. designed the project and wrote the manuscript. T.S., D.L., and S.C. performed experiments and analyzed data.

ACKNOWLEDGMENTS

We thank Shankar Sachidhanandam and Varun Sreenivasan for help with behavioral training. We thank Emmanuel Eggermann and Ramon Reig for advice on electrophysiological recordings. We thank Aurelie Pala and the EPFL Biomicroscopy Center for help with anatomy and imaging. This work was funded by grants from the Swiss National Science Foundation, the European Research Council, and a collaborative grant between the EPFL and the Hebrew University in Jerusalem.

Received: March 19, 2015
Revised: July 8, 2015
Accepted: August 27, 2015
Published: October 1, 2015

REFERENCES

Albin, R.L., Young, A.B., and Penney, J.B. (1989). The functional anatomy of basal ganglia disorders. Trends Neurosci. *12*, 366–375.

Alexander, G.E., and Crutcher, M.D. (1990). Functional architecture of basal ganglia circuits: neural substrates of parallel processing. Trends Neurosci. *13*, 266–271.

Bateup, H.S., Santini, E., Shen, W., Birnbaum, S., Valjent, E., Surmeier, D.J., Fisone, G., Nestler, E.J., and Greengard, P. (2010). Distinct subclasses of medium spiny neurons differentially regulate striatal motor behaviors. Proc. Natl. Acad. Sci. USA *107*, 14845–14850.

Cohen, J.Y., Haesler, S., Vong, L., Lowell, B.B., and Uchida, N. (2012). Neuron-type-specific signals for reward and punishment in the ventral tegmental area. Nature *482*, 85–88.

Cui, G., Jun, S.B., Jin, X., Pham, M.D., Vogel, S.S., Lovinger, D.M., and Costa, R.M. (2013). Concurrent activation of striatal direct and indirect pathways during action initiation. Nature *494*, 238–242.

DeLong, M.R. (1990). Primate models of movement disorders of basal ganglia origin. Trends Neurosci. *13*, 281–285.

Durieux, P.F., Bearzatto, B., Guiducci, S., Buch, T., Waisman, A., Zoli, M., Schiffmann, S.N., and de Kerchove d’Exaerde, A. (2009). D2R striatopallidal neurons inhibit both locomotor and drug reward processes. Nat. Neurosci. *12*, 393–395.

Freeze, B.S., Kravitz, A.V., Hammack, N., Berke, J.D., and Kreitzer, A.C. (2013). Control of basal ganglia output by direct and indirect pathway projection neurons. J. Neurosci. *33*, 18531–18539.

Gerfen, C.R., and Surmeier, D.J. (2011). Modulation of striatal projection systems by dopamine. Annu. Rev. Neurosci. *34*, 441–466.

Gerfen, C.R., Engber, T.M., Mahan, L.C., Susel, Z., Chase, T.N., Monsma, F.J., Jr., and Sibley, D.R. (1990). D1 and D2 dopamine receptor-regulated gene expression of striatonigral and striatopallidal neurons. Science *250*, 1429–1432.

Gerfen, C.R., Paletzki, R., and Heintz, N. (2013). GENSAT BAC Cre-recombinase driver lines to study the functional organization of cerebral cortical and basal ganglia circuits. Neuron *80*, 1368–1383.

Graybiel, A.M., Aosaki, T., Flaherty, A.W., and Kimura, M. (1994). The basal ganglia and adaptive motor control. Science *265*, 1826–1831.

Grillner, S., Hellgren, J., Ménard, A., Saitoh, K., and Wikström, M.A. (2005). Mechanisms for selection of basic motor programs–roles for the striatum and pallidum. Trends Neurosci. *28*, 364–370.

Jin, X., and Costa, R.M. (2010). Start/stop signals emerge in nigrostriatal circuits during sequence learning. Nature *466*, 457–462.

Kravitz, A.V., Freeze, B.S., Parker, P.R., Kay, K., Thwin, M.T., Deisseroth, K., and Kreitzer, A.C. (2010). Regulation of Parkinsonian motor behaviours by optogenetic control of basal ganglia circuitry. Nature *466*, 622–626.

Kravitz, A.V., Tye, L.D., and Kreitzer, A.C. (2012). Distinct roles for direct and indirect pathway striatal neurons in reinforcement. Nat. Neurosci. *15*, 816–818.

Kreitzer, A.C., and Malenka, R.C. (2008). Striatal plasticity and basal ganglia circuit function. Neuron *60*, 543–554.

Kress, G.J., Yamawaki, N., Wokosin, D.L., Wickersham, I.R., Shepherd, G.M., and Surmeier, D.J. (2013). Convergent cortical innervation of striatal projection neurons. Nat. Neurosci. *16*, 665–667.

Madisen, L., Zwingman, T.A., Sunkin, S.M., Oh, S.W., Zariwala, H.A., Gu, H., Ng, L.L., Palmiter, R.D., Hawrylycz, M.J., Jones, A.R., et al. (2010). A robust and high-throughput Cre reporting and characterization system for the whole mouse brain. Nat. Neurosci. *13*, 133–140.

Reig, R., and Silberberg, G. (2014). Multisensory integration in the mouse striatum. Neuron *83*, 1200–1212.

Sachidhanandam, S., Sreenivasan, V., Kyriakatos, A., Kremer, Y., and Petersen, C.C.H. (2013). Membrane potential correlates of sensory perception in mouse barrel cortex. Nat. Neurosci. *16*, 1671–1677.

Schultz, W. (1998). Predictive reward signal of dopamine neurons. J. Neurophysiol. *80*, 1–27.

Schultz, W., Dayan, P., and Montague, P.R. (1997). A neural substrate of prediction and reward. Science *275*, 1593–1599.

Shan, Q., Ge, M., Christie, M.J., and Balleine, B.W. (2014). The acquisition of goal-directed actions generates opposing plasticity in direct and indirect pathways in dorsomedial striatum. J. Neurosci. *34*, 9196–9201.

Shepherd, G.M. (2013). Corticostriatal connectivity and its role in disease. Nat. Rev. Neurosci. *14*, 278–291.

Stephenson-Jones, M., Samuelsson, E., Ericsson, J., Robertson, B., and Grillner, S. (2011). Evolutionary conservation of the basal ganglia as a common vertebrate mechanism for action selection. Curr. Biol. *21*, 1081–1091.

Tai, L.H., Lee, A.M., Benavidez, N., Bonci, A., and Wilbrecht, L. (2012). Transient stimulation of distinct subpopulations of striatal neurons mimics changes in action value. Nat. Neurosci. *15*, 1281–1289.

Tritsch, N.X., and Sabatini, B.L. (2012). Dopaminergic modulation of synaptic transmission in cortex and striatum. Neuron *76*, 33–50.

Wall, N.R., De La Parra, M., Callaway, E.M., and Kreitzer, A.C. (2013). Differential innervation of direct- and indirect-pathway striatal projection neurons. Neuron *79*, 347–360.

Wilson, C.J., and Groves, P.M. (1981). Spontaneous firing patterns of identified spiny neurons in the rat neostriatum. Brain Res. *220*, 67–80.

Xiong, Q., Znamenskiy, P., and Zador, A.M. (2015). Selective corticostriatal plasticity during acquisition of an auditory discrimination task. Nature *521*, 348–351.

Znamenskiy, P., and Zador, A.M. (2013). Corticostriatal neurons in auditory cortex drive decisions during auditory discrimination. Nature *497*, 482–485.

Neuron, Volume *88*

Supplemental Information

Cell-Type-Specific Sensorimotor Processing

in Striatal Projection Neurons

during Goal-Directed Behavior

Tanya Sippy, Damien Lapray, Sylvain Crochet, and Carl C. H. Petersen

Supplemental information

Cell-type-specific sensorimotor processing in striatal projection neurons during goal-directed behavior

Tanya Sippy, Damien Lapray, Sylvain Crochet
and Carl C. H. Petersen

Supplemental Information

Supplemental Figures S1-S4

Supplemental Experimental Procedures

Figure S1

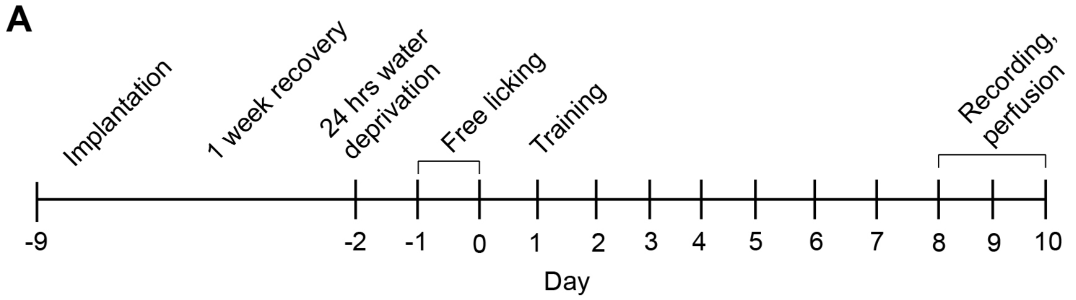

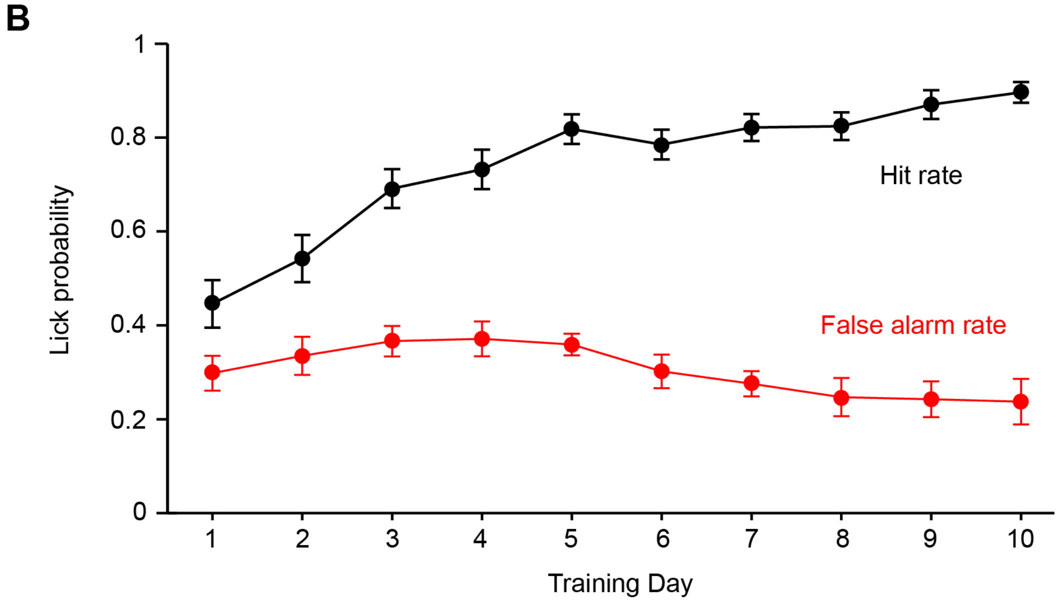

Figure S1. Behavioral training, related to Figure 1.

(A) Timeline of mouse preparation and behavioral training. Mice were implanted with a head-post and allowed to recover for 1 week. They were then water deprived for 24 hours and habituated to head restraint while being given free access to water through the reward spout ('free licking'). On subsequent days, mice were trained on the detection task until stable performance was achieved, usually by training day 8.

(B) Day-by-day behavioral performance curve for all mice (n = 28 mice, of which whole-cell recordings were obtained in 25 of these mice). Mice achieved a stable level of performance after approximately 1 week of training. Values are mean ± SEM.

Figure S2

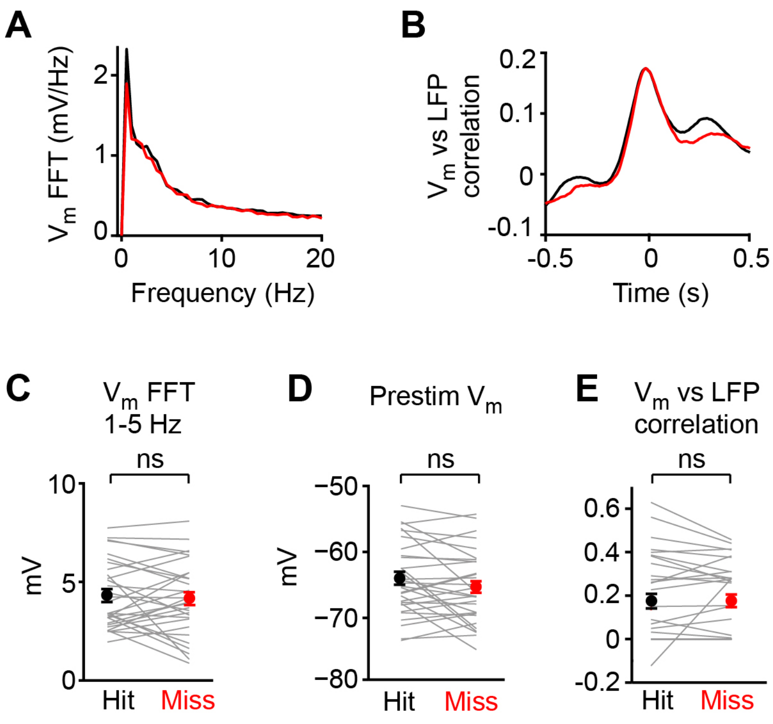

Figure S2. A comparison of pre-stimulus membrane potential revealed no difference between hit and miss trials, related to Figure 2.

(A) Grand average FFT of the V_m calculated for the 2 second period prior to each stimulation for hit (black) and miss (red) trials, averaged across n = 30 cells. Prestimulus V_m displayed prominent slow fluctuations in some striatal neurons, but this was not different in hit *vs* miss trials.

(B) Grand average (n = 21 cells) pre-stimulus cross-correlograms between V_m in striatum and LFP recorded in C2 barrel column for the 2 s preceding both hit (black) and miss (red trials).

(C) The prestimulus V_m FFT 1-5 Hz integral was not significantly different between hit and miss trials (n = 30 cells, Wilcoxon signed rank test p = 0.5).

(D) The prestimulus V_m was not significantly different between hit and miss trials (n = 30 cells, Wilcoxon signed rank test p = 0.5).

(E) The zero-time cross-correlation between striatal V_m and S1 LFP was not significantly different for hit *vs* miss trials (n = 21 cells, Wilcoxon signed rank test p = 0.3).

Values are mean ± SEM. ns = non-significant.

Figure S3

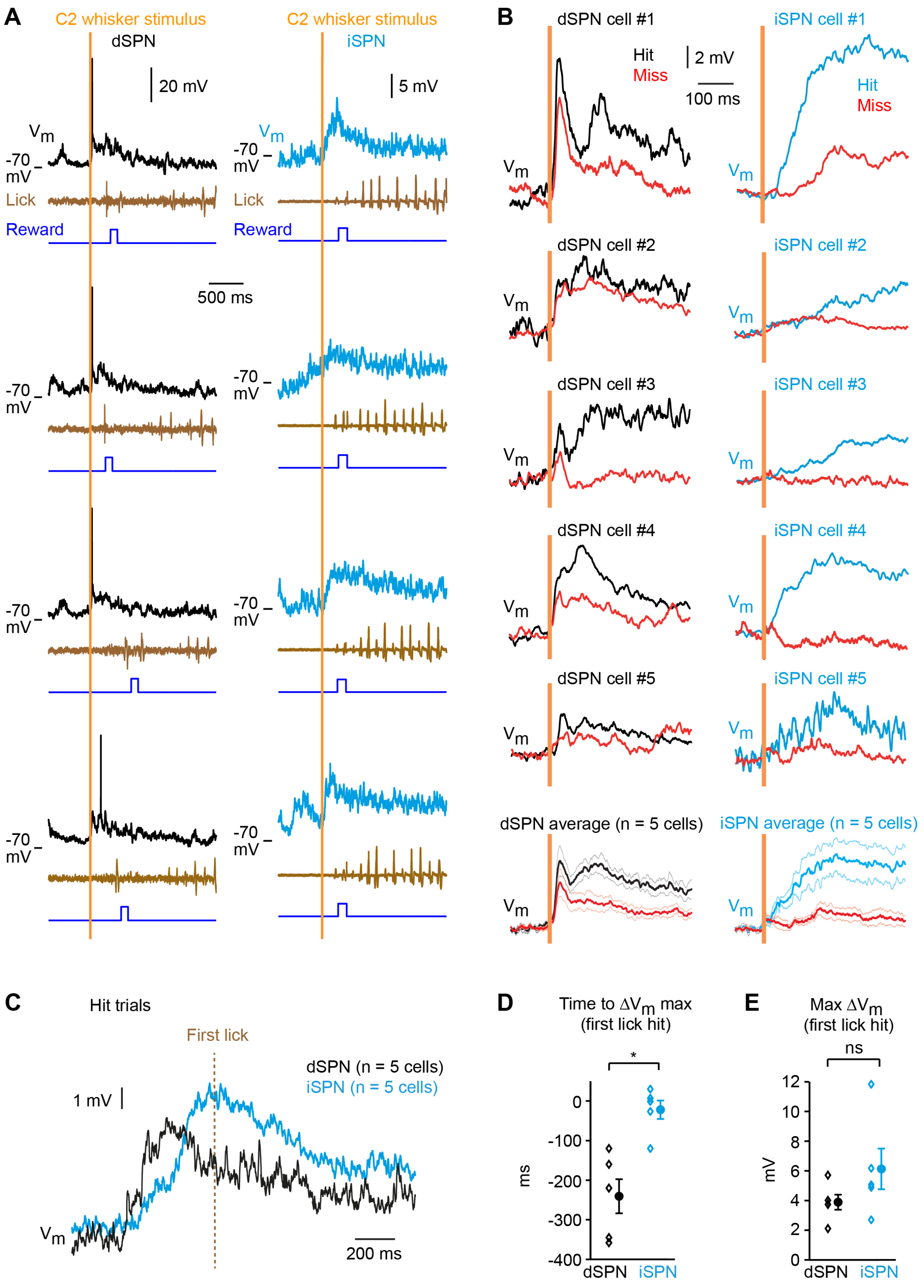

A
C2 whisker stimulus
dSPN
20 mV
Vm
-70 mV
Lick
Reward
C2 whisker stimulus
iSPN
5 mV
500 ms
B
dSPN cell #1
Hit
Miss
2 mV
100 ms
iSPN cell #1
dSPN cell #2
iSPN cell #2
dSPN cell #3
iSPN cell #3
dSPN cell #4
iSPN cell #4
dSPN cell #5
iSPN cell #5
dSPN average (n = 5 cells)
iSPN average (n = 5 cells)
C
Hit trials
First lick
dSPN (n = 5 cells)
iSPN (n = 5 cells)
1 mV
200 ms
D
Time to ΔVm max
(first lick hit)
*
ms
0
-100
-200
-300
-400
dSPN
iSPN
E
Max ΔVm
(first lick hit)
ns
mV
12
10
8
6
4
2
0

Figure S3. Membrane potential of dSPNs and iSPNs, related to Figure 3.

(A) Example V_m traces of 4 individual hit trials (black, left) from the dSPN shown in Figure 3A, showing strong early responses characteristic of this cell-type. Example V_m traces of 4 individual hit trials (blue, right) from the iSPN shown in Figure 3B.

(B) The average hit and miss trial V_m traces for each of the identified dSPNs (hit black, miss red, left) and iSPNs (hit blue, miss red, right). The grand average V_m traces (mean thick traces, SEM lighter traces) across hit and miss trials for the dSPNs (n = 5 cells, hit black, miss red, left) and iSPNs (n = 5 cells, hit blue, miss red, right).

(C) Grand average V_m traces for dSPNs (black, n = 5 cells) and iSPNs (blue, n = 5 cells) showing first lick triggered averages on hit trials. The V_m of dSPNs depolarized earlier than iSPNs before the first lick of hit trials.

(D) The time of the peak V_m depolarization on hit trials around the time of the first lick (t = 0 ms) occurred significantly earlier for dSPNs *vs* iSPNs (Wilcoxon-Mann-Whitney two-sample rank test p = 0.02).

(E) Peak V_m depolarization of traces aligned to the time of the first lick during hit trials compared to baseline V_m was not significantly different between dSPNs and iSPNs (Wilcoxon-Mann-Whitney two-sample rank test p = 0.2).

Values are mean ± SEM. *, $p < 0.05$. ns = non-significant.

Figure S4

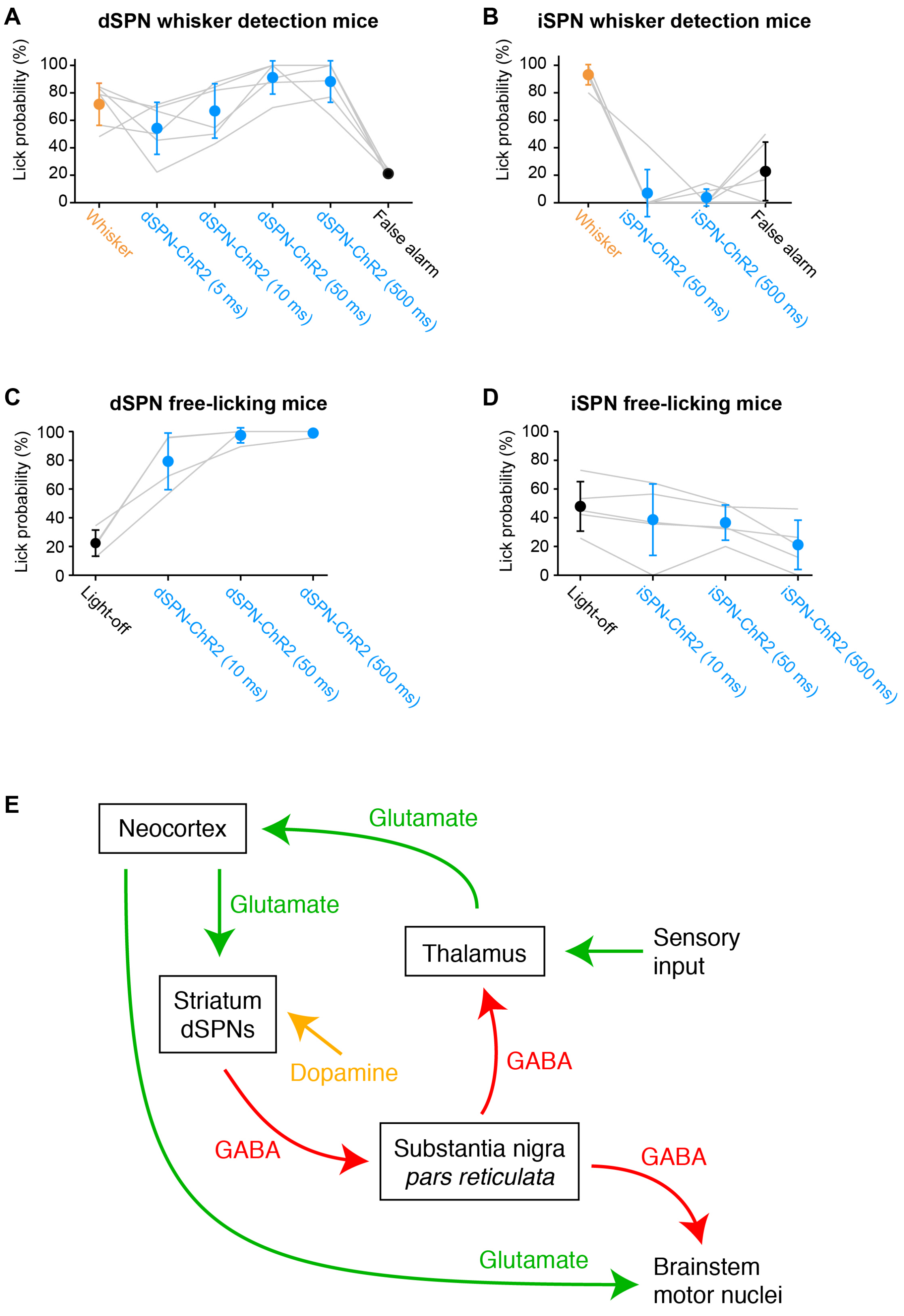
A
dSPN whisker detection mice
Lick probability (%)
100
80
60
40
20
0
Whisker
dSPN-ChR2 (5 ms)
dSPN-ChR2 (10 ms)
dSPN-ChR2 (50 ms)
dSPN-ChR2 (500 ms)
False alarm
B
iSPN whisker detection mice
Lick probability (%)
100
80
60
40
20
0
Whisker
iSPN-ChR2 (50 ms)
iSPN-ChR2 (500 ms)
False alarm
C
dSPN free-licking mice
Lick probability (%)
100
80
60
40
20
0
Light-off
dSPN-ChR2 (10 ms)
dSPN-ChR2 (50 ms)
dSPN-ChR2 (500 ms)
D
iSPN free-licking mice
Lick probability (%)
100
80
60
40
20
0
Light-off
iSPN-ChR2 (10 ms)
iSPN-ChR2 (50 ms)
iSPN-ChR2 (500 ms)
E
Neocortex
Glutamate
Glutamate
Thalamus
Sensory input
Striatum dSPNs
Dopamine
GABA
GABA
Substantia nigra pars reticulata
GABA
Glutamate
Brainstem motor nuclei

Figure S4. Optogenetic stimulation of dSPNs, but not iSPNs, readily substituted for whisker stimulation, related to Figure 4.

(A) The probability that a mouse trained in the whisker detection task will lick the reward spout (lick probability) is plotted *vs* various stimulation parameters. For dSPN-ChR2 mice, blue light stimulation, ranging from 5-500 ms in duration could effectively substitute for whisker stimulation. Performance of the mice with even the shortest light pulse used, 5 ms, was not statistically significantly different from whisker stimulation (n = 6 mice, Kruskal-Wallis test followed by Student-Newman-Keuls test $p > 0.05$) and with longer duration (50 and 500 ms) mice performed even better than with whisker stimulation (n = 6 mice, Kruskal-Wallis test followed by Student-Newman-Keuls test $p < 0.01$). Values are mean ± SD.

(B) For iSPN-ChR2 mice trained in the whisker detection task, blue light stimulation of 50 ms or 500 ms duration could not substitute for whisker stimulation, and performance in response to light stimulation in these animals was not different from the false alarm rate (n = 6 mice, Kruskal-Wallis test followed by Student-Newman-Keuls test $p > 0.05$). Values are mean ± SD.

(C) In order to investigate a striatal motor signal in the absence of training in the whisker detection task, we trained thirsty mice to lick a reward spout, and water was delivered anytime the mouse licked after a 4 s period without any lick. Random unrewarded optogenetic stimulation of dSPNs readily evoked licking in these highly motivated free-licking mice. Licking probability was significantly higher following blue light pulses of any duration (10 ms, 79.3 ± 19.8 %; 50 ms, 97.4 ± 5.3 %; or 500 ms, 98.9 ± 2.2 %) than in the absence of light pulse (Light-off, 22.4 ± 9.1 %) (n = 4 mice, Kruskal-Wallis test followed by Student-Newman-Keuls test $p < 0.01$). Values are mean ± SD.

(D) Optogenetic stimulation of iSPNs in free-licking mice did not evoke licking. Licking probability was not significantly different following blue light pulses of any duration (10 ms, 38.7 ± 24.9 %; 50 ms, 36.6 ± 12.3 %; or 500 ms, 21.2 ± 17.1 %) than in the absence of light pulse (Light-off, 47.9 ± 17.3 %) (n = 5 mice, Kruskal-Wallis test, $p > 0.05$). Values are mean ± SD.

(E) Schematic diagram illustrating pathways likely to contribute to the goal-directed sensorimotor transformation underlying the whisker detection task. Ascending somatosensory glutamatergic input from the brainstem brings

whisker sensory information to the thalamus. Thalamocortical neurons signal information to the neocortex. In a previous study we found that primary somatosensory neocortex participates causally in performance of the detection task (Sachidhanandam et al., 2013). The neocortex innervates many brain regions involved in motor control, including a strong projection to the striatum. The data in this study are consistent with direct-pathway striatal projection neurons (dSPNs) sending a fast transient inhibitory signal to the substantia nigra *pars reticulata* in response to detected whisker stimuli. Transient inhibition of GABAergic neurons in substantia nigra *pars reticulata* could then mediate disinhibition of thalamus and brainstem motor nuclei. The resulting enhanced activity in thalamus and brainstem motor nuclei could contribute to driving the learned goal-directed sensorimotor transformation underlying the whisker detection task.

Supplemental Experimental Procedures

Animal preparation and surgery

All experiments were carried out with 5-9 week old male and female mice in accordance with the Swiss Federal Veterinary Office (authorization 1628.3). D1-Cre, A2A-Cre and D2-Cre bacterial artificial chromosome (BAC) transgenic mice were obtained from Gene Expression Nervous System Atlas (GENSAT; founder line EY262 and EY217 for D1-Cre, KG139 for A2A-Cre and ER44 for D2-Cre), and purchased through the Mutant Mouse Regional Resource Centers (MMRRC). These mice were then crossed with lox-stop-lox (LSL) tdTomato reporter mice (B6.Cg-Gt(ROSA)26Sor$^{tm9(CAG-tdTomato)Hze/}$J) to obtain D1-Cre x LSL-tdTomato, A2A-Cre x LSL-tdTomato or D2-Cre x LSL-tdTomato mice that were implanted with a light-weight metal head-post and a recording chamber under ketamine/xylazine anesthesia. Three to seven days after surgery, all whiskers were trimmed on both sides except the C2 whiskers. Intrinsic signal optical imaging was carried out to locate the C2 barrel column in the left hemisphere.

Behavioral Training

One week after implantation, all whiskers were again trimmed except the C2 whisker on either side. Over a period of 1-2 days mice were adapted to head restraint. They were subsequently water deprived for 24 hours before behavioral training. During training in the detection task, mice received water exclusively in the behavioral setup, and were allowed brief free access (15 minutes) to wet food immediately thereafter in an individual cage. Iron filings were applied to the right C2 whisker at the beginning of each training session, allowing the whisker to be vertically deflected by a 1 ms current pulse passed through an electromagnetic coil placed immediately beneath the head of the mouse. Mice were then trained to associate this deflection of the C2 whisker with the availability of water at a reward spout placed within reach of their tongue. If the mouse licked the spout within the reward window (1 s), it was considered a hit trial, and the mouse received a drop of water. If not, it was considered a miss trial and no reward was delivered. Whisker stimuli were delivered without any preceding cues at random time intervals ranging

between 6 and 10 seconds. To discourage spontaneous licking, a 4 s timeout period was imposed during which no lick should occur in order to start a trial. Trials with whisker stimuli were randomly interleaved with ‘catch trials’ in which no stimulus was given. If licks occured during the response window of a catch trial it was considered a false alarm.

Mice were able to achieve a stable level of performance over the course of a few days (Figure S1), with a high hit rate and a low false alarm rate. Ambient white noise was played at all times to mask any potential auditory cues arising from whisker stimulation. Licks were detected with piezo-film attached to the reward spout. Behavioral control and data collection were carried out with custom written computer programs using either an ITC18 (Instrutech) interfaced through IgorPro (Wavemetrics) or a National Instruments board interfaced through LabView. Once the mice achieved a consistent level of performance (hit rate greater than 80% and false alarm rate less than 35%) they were considered adequately trained and were subsequently used for electrophysiological recordings or optogenetic manipulations.

The state of motivation of the mice plays an important role in determining the probability of licking in response to whisker stimulation. However, within the relatively short V_m recording periods we did not find any change in behavioral performance. The ‘miss rate’ of 49.7 ± 5.7 % (i.e. ‘hit rate’ of 50.3 ± 5.7 %) at the beginning of the recordings (first quarter of trials) did not differ appreciably from the ‘miss rate’ of 40.7 ± 6.3 % (i.e. ‘hit rate’ of 59.3 ± 6.3 %) at the end of the recordings (last quarter of the trials).

Electrophysiology

On the day of recording, a small (less than 1 mm diameter) craniotomy was made under isoflurane anesthesia over the dorsolateral striatum (stereotaxic coordinates: 0 mm anterior and 2.8-3.0 mm lateral of bregma). Mice were allowed to recover from anesthesia for two to four hours. Then, whole-cell patch-clamp recordings were obtained as previously described (Sachidhanandam et al., 2013; Reig and Silberberg, 2014). 6-8 MΩ glass pipettes were filled with a solution containing (in mM): 135 potassium gluconate, 4 KCl, 10 HEPES, 10 sodium phosphocreatine, 4 MgATP, 0.3

Na_3GTP (adjusted to pH 7.3 with KOH), to which 2-4 mg/ml biocytin was added. In some experiments, local field potential (LFP) was recorded with a 2-4 MΩ glass pipette filled with Ringer solution and lowered to a depth of 150-250 µm from the pia in the C2 barrel column. V_m and LFP signals were amplified using a Multiclamp 700B amplifier (Axon Instruments), Bessel filtered at 10 kHz, and digitized at 20 kHz by an ITC-18 (Instrutech Corporation) under the control of IgorPro (Wavemetrics). All patch-clamp recordings were obtained in current-clamp mode without injection of any current, except during the characterization of intrinsic electrophysiological properties. V_m was not corrected for liquid junction potentials.

At the start of each recording, a series of increasing current steps was injected into each neuron. We proceeded with the recording if the neuron displayed both a stable resting V_m and overshooting action potentials. The series resistance (also termed access resistance) of the recordings ranged from 25-40 MΩ. On average across recordings, our measurements of V_m in SPNs during task performance included 19.1 ± 2.1 hit trials and 12.6 ± 1.8 miss trials.

Optogenetic activation

D1-Cre (strain EY217) mice and A2A-Cre mice (4 week old male and females) were injected under isoflurane anesthesia with an adenoassociated virus (AAV) serotype 5 expressing double floxed inverted reading frame humanized ChR2 (H134R) fused to YFP under control of EF1α promoter (AAV2/5 DIO-EF1α-hChR2(H134R)-eYFP; virus made by Penn Vector Core). Prior to virus injection a ~0.5 mm craniotomy was made over the area of dorsolateral striatum (stereotaxic coordinates: 0 mm anterior and 2.6 mm lateral to bregma). A glass injection pipette was tip filled with the virus solution and lowered into the dorsolateral striatum. 200 nl of the virus solution was slowly injected at a depth of 2500 µm below the pia with a flow rate of 40-50 nl/min. The micropipette was left in position for 8-10 minutes and then slowly retracted to prevent backflow of virus along the shaft.

Mice expressing ChR2 were trained in the whisker detection task as described above, with the addition of ambient blue light. On the transfer test day, a craniotomy of ~1 mm diameter was opened over the dorsolateral

striatum, and animals were allowed to recover for at least 2 hours. A multimode fiber optic cable (Thorlabs; 300 μm) coupled to a 473 nm blue laser (GMP) was then lowered to a depth of ~2 mm into the brain directly above dorsolateral striatum. During behavioral testing, a third kind of trial (ChR2 stimulus trials) was randomly interleaved with whisker stimulus and catch trials. The light stimulus consisted of a single pulse of blue light of either 5, 10, 50 or 500 ms. At the end of the experiment, the mice were anesthetized and perfused so their brains could be recovered to verify both the injection sites and placement of the optic fiber.

We also carried out optogenetic stimulation experiments in free-licking mice, which were not trained in the whisker detection task, but were water-deprived and trained to lick the reward spout in order to obtain water rewards (Figure S4). Three weeks following virus injection, mice were water deprived and trained in a free-licking task where water was delivered any time the mouse would lick after a 4 s period of no licking. Mice were trained for 2-4 sessions. On the test day, an optical fiber was lowered to a depth of ~2 mm into the brain directly above dorsolateral striatum. During behavioral testing, we randomly applied light stimuli of 10, 50 or 500 ms, or no light pulse (Light-off). Licking in response to optogenetic stimuli was not reinforced by water rewards. Lick probability was computed within a 1 s time window following the onset of the optogenetic stimulus.

Histology and immunohistochemistry

After recording, the mice were perfused with a 4% paraformaldehyde (PFA) in 0.1 M PBS. Brains were post-fixed for maximum 24 hours in the same solution, which was then replaced by a 0.1 M PBS solution. 100 μm-thick coronal sections were cut using a semi-automated vibratome (VT1000S, Leica). Streptavidin coupled to Alexa 647 (1:2000, Invitrogen) was used to reveal biocytin filling of postsynaptic neurons. Images were obtained with a laser scanning confocal microscope (LSM 700, Zeiss) equipped with an oil-immersion 63x/1.4NA objective. Three-dimensional anatomical reconstructions were traced from confocal fluorescence image stacks using Neurolucida (MBF Bioscience).

Data analysis

All data analysis was performed in MATLAB using custom written algorithms. To assess the amplitude of the early sensory response, the maximum depolarization of V_m over 0-50 ms post stimulation was calculated and subtracted from the baseline (calculated to be the average V_m over the 500 ms before the stimulus). To measure the amplitude of the late depolarization, V_m was averaged across 50-250 ms post stimulus and the value subtracted from the baseline (the average V_m over the 500 ms before the stimulus). Fast Fourier Transforms (FFTs) were calculated for 2 second segments immediately prior to each whisker stimulus or catch trial. The amplitude of low frequency V_m fluctuations was calculated by integrating the calculated FFTs from 1-5 Hz. We used the first and second derivative of the membrane potential to calculate response onset. Slopes were obtained as $\Delta V/\Delta t$ between onset and $\Delta V max$ time interval.

The effects of spontaneous unrewarded licking upon striatal V_m was quantified across licking bouts that occurred at least 4 seconds prior to, and least 4 seconds after any whisker stimulation. In addition each spontaneous licking bout was separated by at least 2 seconds from the previous licking bout. The first lick in the licking bout was identified, and used to align epochs before averaging.

All values are presented as mean ± SEM (except Figures 4D and S4A-D, which show mean ± SD). Non-parametric statistical tests were used to assess significant differences. The Wilcoxon-Mann-Whitney two-sample rank test was used for unpaired samples (dSPNs *vs* iSPNs). The Wilcoxon signed rank test was used for paired samples (hit *vs* miss trials). For multiple comparisons, we first performed a Kruskal-Wallis test, then a nonparametric variation on the Student-Newman-Keuls test to compare between samples.
